# MPXV H3L elicits broadly directed CD4 T cell responses in mpox patients and MVA vaccinees

**DOI:** 10.1038/s44298-026-00205-5

**Published:** 2026-07-07

**Authors:** David da Fonseca Araújo, Christoph Schultheiß, Leon Cords, Hendrik Karsten, Robin Woost, Sonja Grobecker, Tim Westphal, Liz Lam, Marc Lütgehetmann, Susanne Pfefferle, Olaf Degen, Sven Peine, Hanna Matthews, Stefan Schmiedel, Guido Schäfer, Hanna-Marie Weichel, Marylyn Addo, Alessandro Sette, John Sidney, Mascha Binder, Christian Hoffmann, Julian Schulze zur Wiesch

**Affiliations:** 1 Infectious Diseases Unit, 1. Department of Medicine, University Medical Center Hamburg-Eppendorf, Hamburg, Germany; 2 Department of Biomedicine, University and University Hospital Basel, Basel, Switzerland; 3 German Center for Infection Research (DZIF), Partner Site Hamburg-Lübeck-Borstel-Riems, Hamburg, Germany; 4 Institute for Medical Microbiology, Virology and Hygiene, University Medical Center Hamburg-Eppendorf, Hamburg, Germany; 5 Institute of Transfusion Medicine, University Medical Center Hamburg-Eppendorf, Hamburg, Germany; 6 ICH, Hamburg, Germany; 7 IIRVD—Institute for Infection Research and Vaccine Development, University Medical Center Hamburg-Eppendorf, Hamburg, Germany; 8 Center for Infectious Disease and Vaccine Research, La Jolla Institute for Immunology (LJI), La Jolla, CA USA

**Keywords:** Diseases, Immunology

## Abstract

Data on MPXV-specific T-cell responses at the single-peptide level remain limited in patients with mpox and in MVA-BN vaccinees. Here, we characterized the breadth and specificity of MPXV-specific T-cell responses at the single-peptide level after in vitro expansion with overlapping 20-mer peptides spanning the MPXV proteins H3L, A35R, and B6R. The study included 28 adult males: 15 with a history of mpox (including 6 with additional MVA-BN vaccination), 7 MVA-BN-only vaccinees, and 6 unexposed participants. All mpox and MVA-BN participants responded to at least one H3L peptide, indicating broad immunogenicity, while responses to A35R and B6R were more common in the mpox group. Notably, the breadth of B6R-specific CD4^+^ T-cell responses correlated with hybrid immunity (*r* = 0.6; *p* = 0.02). Interestingly, MVA-BN and mpox individuals demonstrated distinct immunodominant patterns: H3L_251–270 and H3L_211–230 were mainly recognized among mpox individuals, whereas MVA-BN recognized H3L_221–240 more frequently. High-affinity HLA binding to multiple H3L peptides suggests broad population coverage. Additional immunogenetic analysis revealed a shared TRBV15 clonotype in about 50% of mpox cases. In summary, these findings highlight H3L as a potential vaccine target, guiding the development of next-generation multi-antigen MPXV vaccines to elicit comprehensive T-cell immunity.

## Introduction

Mpox is a zoonotic disease caused by the mpox virus (MPXV), with an incubation period of 5–21 days and typically presenting with fever, lymphadenopathy, and a characteristic exanthematous rash^1,2^. MPXV belongs to the genus Orthopoxvirus within the Poxviridae family^3^, which also includes vaccinia virus (VACV) and variola virus (VARV). The successful eradication of smallpox through VACV-based vaccination campaigns was facilitated by the high degree of protein homology among members of the poxvirus family^4,5^.

Phylogenetically, MPXV comprises two main clades, with two additional subclades in Clade II: Clade I (Central African), which is associated with higher virulence, and Clades IIa and IIb (West African), which are less virulent^6^. The 2022 global outbreak, caused by lineage B.1 of Clade IIb^7^, prompted the WHO to declare mpox a public health emergency and led to the widespread use of the third-generation live attenuated MVA-BN smallpox vaccine, which has shown 75–86% effectiveness^8^. MPXV is a large dsDNA virus (197 kB genome, approximately 190 ORFs) that exists in two infectious forms: extracellular enveloped virions (EEV) for cell-to-cell spread and intracellular mature virions (IMV) for host-to-host transmission^9,10^.

A recent study demonstrated significant T-cell cross-reactivity between VACV and MPXV by analyzing previously identified orthopoxvirus epitopes from the Immune Epitope Database (IEDB) and predicting potential MPXV epitopes^11^. Although in silico analyses have predicted at least 70% conservation of virus-specific CD4⁺ epitopes and at least 80% conservation of CD8⁺ epitopes, comprehensive in vivo data on MPXV-specific T-cell responses at the individual peptide level remain limited. Despite high orthopoxvirus homology, amino acid differences at key positions may influence epitope recognition, as MVA-based vaccines retain major MPXV orthologs but have genomes that are approximately 14% shorter^12^. Given the critical role of T-cell responses in orthopoxvirus control^13^, a thorough characterization of MPXV-specific epitopes beyond overall reactivity patterns is essential for understanding protective immunity.

In this study, we comprehensively analyzed the breadth and specificity of MPXV-specific CD4^+^ T-cell responses at the single peptide level using overlapping 20-mer peptide sets spanning three MPXV surface proteins: H3L, A35R, and B6R. These proteins share more than 90% sequence identity with MVA and MPXV and elicit robust B- and T-cell responses after infection and vaccination, playing crucial roles in viral neutralization, immune control, and viral dissemination^14–19^. To cover both infectious forms of the virus, H3L was selected as a representative protein of IMV, while A35R and B6R represent the EEV, enabling a comprehensive assessment of MPXV-specific T-cell responses throughout the viral life cycle^20^. H3L serves as the primary attachment protein of IMV, mediating viral entry by binding to cell-surface heparan sulfate through its N-terminal α-helical domain^21^. Beyond its essential role in viral adhesion, H3L exhibits exceptional immunogenicity; MPXV-infected individuals show significantly higher anti-H3L antibody responses than those of VACV-vaccinated individuals^15^. A35R functions as a dimeric envelope protein of EEV, facilitating antibody-resistant viral cell-to-cell spread and playing a crucial role in viral dissemination by forming actin-containing microvilli^22^. This protein consistently emerges as one of the most immunogenic MPXV antigens, as mpox recoverees display significantly higher anti-A35R antibody levels than those against other MPXV antigens, which strongly correlates with VACV neutralization^15^. Additionally, studies have demonstrated that A35R and its orthologs are important virulence factors in orthopoxviruses, as the loss of A35R results in increased attenuation^23,24^. B6R, a palmitoylated glycoprotein and part of the EEV, is required for efficient cell-to-cell spread by dissolving the outer membrane and mediating viral exit; it also contributes to complement control^25^.

To thoroughly examine the immune recognition patterns of these proteins, we systematically compared virus-specific T-cell responses to H3L, A35R, and B6R among individuals with recent or acute mpox and MVA-BN vaccinees, incorporating known HLA backgrounds. This comparison aimed to identify key immunogenic epitopes and clarify differences in immune responses, offering a deeper understanding of MPXV-specific T-cell immunity. The epitope-level analysis addresses important gaps in our knowledge and highlights potential antigenic targets for next-generation vaccine development.

## Results

### Clinical and demographic characteristics of the study cohort

Clinical and demographic characteristics of the study participants are summarized in Table 1 and Supplementary Table 1. The exclusively male cohort comprised (A) individuals with prior or acute MPXV infection (mpox group, *n* = 15), (B) uninfected individuals vaccinated with at least two doses of MVA-BN (MVA-BN group, *n* = 7), and (C) individuals with neither prior MPXV infection nor MVA-BN vaccination (unexposed group, *n* = 6).

**Table 1:** Clinical characteristics of study participants

	Mpox (*n* = 15)	MVA-BN (*n* = 7)	Unexposed (*n* = 6)
Age, mean (range)	42 (25–66)	41 (22–64)	24 (23–25)
Sex, %			
Male	100	100	100
Female	0	0	0
HIV coinfection			
HIV-positive, *n*	5	1	0
CD4^+^ T cells, cells/µL, mean (range)	766 (343–1529)	na	–
On ART, *n*	5	1	–
MVA-BN vaccination status			
Unvaccinated	9	0	6
1 dose	3	0	0
2 doses	3	7	0
Days since last antigen exposure, mean (range)	338 (0–481)	257 (105–368)	–

Demographic and clinical characteristics of individuals with mpox (*n* = 15), MVA-BN vaccinees (*n* = 7), and unexposed controls (*n* = 6) are shown. Variables include age, sex, HIV status with CD4⁺ T-cell counts for HIV-positive participants, antiretroviral therapy (ART), MVA-BN vaccination status, and days since last antigen exposure. CD4⁺ T-cell count was unavailable for one HIV-positive MVA-BN vaccinee; one mpox participant was excluded from the time-since-exposure calculation due to a missing diagnosis date.

Among the mpox group, 14 individuals had clinically resolved the MPXV infection, with a mean time of 338 days from PCR-confirmed diagnosis to study inclusion. Only one participant had an acute MPXV infection at the time of recruitment. In the MVA-BN group, the mean time since the last vaccination was 257 days. Six individuals within the mpox group also received MVA-BN vaccination with varying regimens: Two individuals received one dose, three received two doses before infection—averaging 213 days before mpox diagnosis—and one participant received a single vaccine dose 44 days after recovery. Notably, four mpox individuals and one MVA-BN vaccinee were HIV⁺. All were on antiretroviral therapy and had stable CD4⁺ T-cell counts ≥200 cells/µL (mean = 766) at the time of analysis.

### H3L, A35R, and B6R-specific peptides elicit broadly directed IFN-γ^+^ T-cell responses in mpox and MVA-BN-vaccinated individuals

**Fig. 1:**
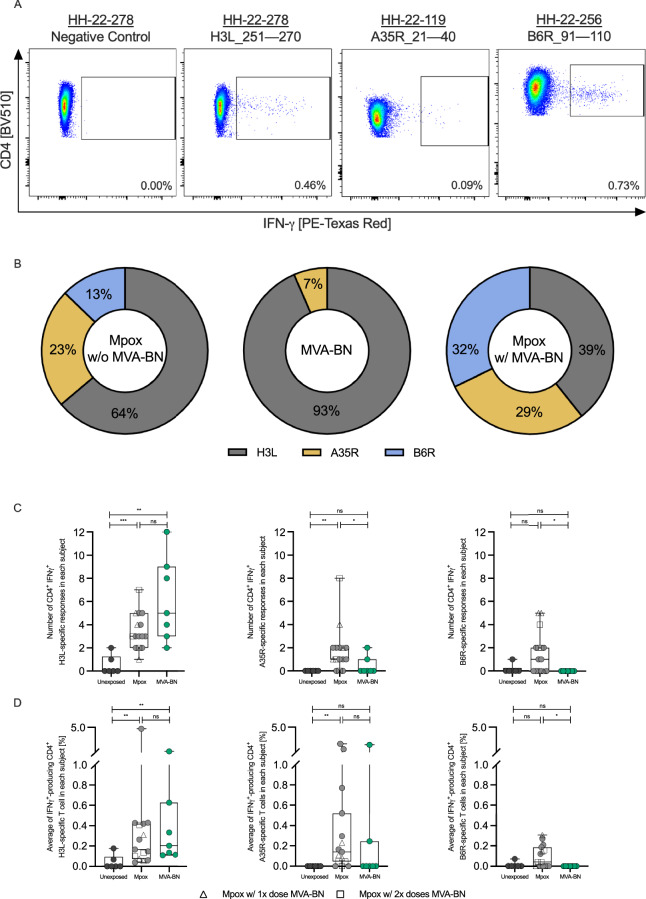
MPXV-specific CD4⁺ T-cell responses to glycoproteins H3L, A35R, and B6R following infection and vaccination. **A** Representative flow cytometry plot showing MPXV-specific CD4⁺ T-cell responses from three mpox donors upon peptide stimulation. From left to right: donor HH-22-278 (negative control and H3L_251–270), donor HH-22-119 (A35R_21–40), and donor HH-22-256 (B6R_91–110). **B** Distribution of responses across the MPXV glycoproteins H3L (gray), A35R (yellow), and B6R (blue), expressed as the percentage of total peptide-specific responses within each group: mpox without MVA-BN vaccination (*n* = 9), MVA-BN (*n* = 7), and hybrid mpox with MVA-BN vaccination (*n* = 6). Statistical analyses were conducted using the Mann–Whitney *U* test; significance levels are indicated as follows: *p < 0.05, **p < 0.01, ***p < 0.001, and ns = not significant. **C** Breadth of MPXV-specific CD4⁺ T-cell responses (number of reactive peptides per individual) for H3L (left), A35R (middle), and B6R (right) in unexposed controls (*n* = 6), mpox (*n* = 15), and MVA-BN–vaccinated donors (*n* = 7). Individuals with hybrid immunity (rMPXV + MVA-BN) are indicated by triangles (one dose of MVA-BN) and squares (two doses). **D** Magnitude of MPXV-specific CD4⁺ T-cell responses (mean frequency of IFN-γ-producing CD4⁺ T cells among total CD4⁺ T cells) for the same groups and glycoproteins as in (**B**).

Virus-specific CD4⁺ T-cell responses were measured by IFN-γ ELISpot after in vitro expansion, stimulated with overlapping 20-mer peptides covering the MPXV proteins H3L, A35R, and B6R. These results were confirmed by ICS for IFN-γ following restimulation with the corresponding MPXV-specific single peptide (Representative FACS plots, Fig. 1A)^26–29^.

We identified 149 individual MPXV peptides that induced IFN-γ^+^ CD4^+^ T-cell responses across all 22 infected and vaccinated individuals analyzed. The number of peptide-specific responses per individual was similar, with a mean of 6.9 responses in the mpox group and a slightly lower average of 6.6 responses across MPXV proteins in the MVA-BN group. MPXV-specific CD4^+^ T-cell responses to H3L, A35R, and B6R were evenly distributed among mpox individuals who also received the MVA-BN vaccination (about 33% on average, Fig. 1B). In contrast, MPXV-specific CD4^+^ T-cell responses in unvaccinated mpox individuals were mostly directed toward H3L (64%), with fewer responses targeting A35R (23%) and B6R (13%). Notably, MPXV-specific CD4^+^ T-cell responses in the MVA-BN group were almost exclusively directed against H3L (93%), with only 7% recognizing A35R and none recognizing B6R.

### H3L represents a major immunogenic target for virus-specific CD4⁺ T-cell responses in both mpox and MVA-BN-vaccinated individuals

Most mpox participants exhibited at least one detectable virus-specific CD4^+^ T-cell response to A35R (12/15) and B6R (9/15), whereas MVA-BN vaccinees showed fewer responses to A35R (2/7) and none to B6R. Notably, all mpox individuals and MVA-BN participants responded to at least one H3L-derived peptide (Fig. 1C). This pattern remained consistent after stratifying mpox individuals by prior vaccination history; however, mpox individuals with prior MVA-BN vaccination tended to have more B6R-specific CD4^+^ T-cell responses (mean = 3) than those without hybrid immunity (mean = 0.67; Supplementary Fig. 1). Of note, low-level H3L- and B6R peptide-specific CD4^+^ T-cell responses were observed in three unexposed subjects following restimulation.

To indicate antigen-specific activation, we measured the magnitude of MPXV-specific CD4^+^ T-cell responses by calculating the average percentage of IFN-γ⁺ cells for each peptide within the ICS assay. The magnitude of H3L-specific responses was similar between mpox (mean = 0.52%) and MVA-BN-vaccinated individuals (mean = 0.39%), whereas the A35R-specific responses were slightly higher in the mpox group (Fig. 1D). Finally, we compared the breadth and magnitude of MPXV-specific CD4^+^ T-cell responses between HIV^+^ (*n* = 5) and HIV^−^ (*n* = 10) mpox participants but found no significant differences across any of the tested antigens (Supplementary Fig. 2A, B).

### Comparable breadth of MPXV-specific CD8⁺ T-cell responses upon stimulation with H3L, A35R, and B6R 20-mer peptides

Although the detection of MPXV-specific CD4^+^ T-cell responses is favored by stimulation with 20-mer peptides, we did not initially exclude analysis of CD8^+^ T-cell responses. Among the 22 mpox and MVA-BN-vaccinated individuals, we identified 100 individual MPXV peptides that induced IFN-γ^+^ CD8^+^ T-cell responses: 74 in mpox patients (average = 4.9) and 26 in MVA-BN vaccinees (average = 3.7); none were detected in unexposed controls.

Overall, H3L, A35R, and B6R peptide-specific CD8^+^ T-cell responses were significantly lower than those observed for CD4^+^ T-cell responses (Supplementary Fig. 3). Vaccinees generally showed a higher number of H3L peptide-specific CD8^+^ T-cell responses per individual (average = 3.2) compared to mpox patients (average = 1.9; Supplementary Fig. 4A). Conversely, A35R- and B6R-specific CD8^+^ T-cell responses were more common in the mpox group. While the breadth of virus-specific CD8^+^ T-cell responses against H3L and A35R was largely similar between the groups, that of B6R-specific responses was higher in the mpox group (Supplementary Fig. 4B).

Spearman correlation analyses showed a positive correlation between antigen exposure count (total number of exposures to MPXV and/or MVA-BN) and B6R-specific CD4^+^ T-cell breadth (*r* = 0.6; *p* = 0.02), as well as between the antigen exposure count and the breadth and magnitude of H3L- and A35R-specific CD8^+^ T-cell responses (Supplementary Fig. 4D). Conversely, B6R peptide-specific CD4^+^ (*r* = −0.57; *p* = 0.038) and CD8^+^ (*r* = −0.82; *p* = 0.0005) response breadths negatively correlated with the time since diagnosis.

Taken together, virus-specific CD4⁺ and CD8⁺ T-cell responses to MPXV antigens exhibited generally similar reactivity patterns, with H3L emerging as a prominent immunogenic target that was most pronounced in the CD4⁺ compartment and further boosted by MVA-BN vaccination, while A35R and B6R responses were more commonly seen after natural infection.

### Frequently detected H3L-specific CD4⁺ T-cell responses define immunodominant targets in mpox-infected individuals

**Fig. 2:**
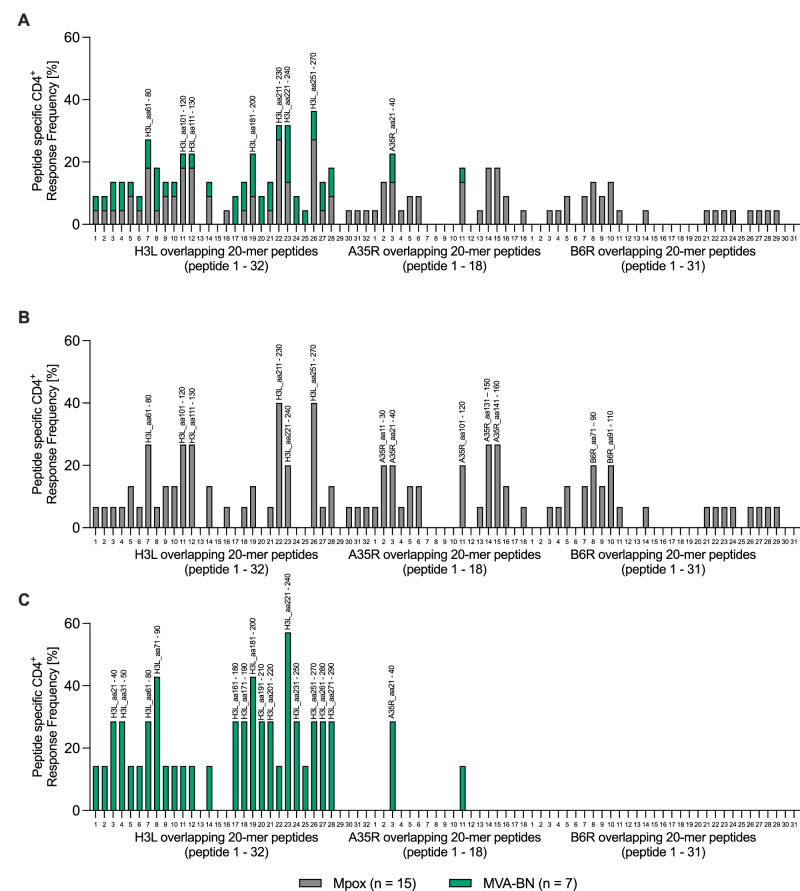
Mapping of MPXV-specific CD4⁺ T-cell responses to overlapping 20-mer peptides of H3L, A35R, and B6R. **A** Frequency of MPXV-specific CD4⁺ T-cell responses to each peptide among all analyzed individuals (*n* = 22; including mpox and MVA-BN–vaccinated individuals). **B** Response frequencies in mpox participants only (*n* = 15). **C** Response frequencies in MVA-BN–vaccinated donors only (*n* = 7). Each bar represents the percentage of individuals exhibiting an MPXV-specific CD4⁺ T-cell response to a specific 20-mer peptide (with 10 amino acid overlaps) derived from the MPXV glycoproteins H3L (32 peptides), A35R (18 peptides), and B6R (31 peptides). Peptides recognized by 20% or more of subjects are labeled with their respective peptide number and amino acid range.

The eight most frequently detected peptides with response rates of ≥20% among all infected and vaccinated study participants (*n* = 22) are listed in Table 2 and shown in Fig. 2. To further characterize these immunodominant regions, Table 3 provides a comparative sequence analysis highlighting specific amino acid substitutions between MPXV and other OPXVs. Seven peptides were located within H3L, and one within A35R. The peptides H3L_251-270 and H3L_211-230 were most frequently recognized in mpox patients (6/15), whereas the H3L_221-240 peptide was the most common target in MVA-BN vaccinees (4/7). Within the A35R peptide pool, both the mpox (3/15) and MVA-BN (2/7) cohorts mounted a CD4^+^ T-cell response most often toward A35R_21-40. Supplementary Fig. 5 and Supplementary Table 2 depict the respective analysis of CD8^+^ T-cell responses.

**Table 2:** CD4^+^ T-cell response frequencies to commonly detected MPXV-specific peptides

All participants (mpox + MVA-BN, *n* = 22)	Peptide sequence																				RF
H3L-derived peptides (≥20% responders)																					
aa61–80	D	Y	V	F	I	Q	W	T	G	G	N	I	R	D	D	D	K	Y	T	H	32%
aa71–90	N	I	R	D	D	D	K	Y	T	H	F	F	S	G	F	C	N	T	M	C	27%
aa101–120	H	L	A	L	W	D	S	K	F	F	T	E	L	E	N	K	N	V	E	Y	23%
aa111–230	T	E	L	E	N	K	N	V	E	Y	V	V	I	I	E	N	D	N	V	I	27%
aa181–200	Y	D	V	S	L	S	A	Y	I	I	R	V	T	T	A	L	N	I	V	D	23%
aa211–230	G	F	Y	F	E	I	A	R	I	E	N	E	M	K	I	N	R	Q	I	M	32%
aa221–240	N	E	M	K	I	N	R	Q	I	M	D	N	S	A	K	Y	V	E	H	D	32%
aa251–270	T	M	K	P	N	F	W	S	R	I	G	T	V	A	A	K	R	Y	P	G	41%
aa271–290	V	M	Y	T	F	T	T	P	L	I	S	F	F	G	L	F	D	I	N	V	23%
A35R-derived peptides (≥20% responders)																					
aa21–40	C	T	V	P	T	M	N	N	A	K	L	T	S	T	E	T	S	F	N	D	27%

Mpox (*n* = 15)	Peptide sequence																				RF
H3L-derived peptides (≥20% responders)																					
aa41–60	E	V	M	Q	E	K	R	D	V	V	I	V	N	D	D	P	D	H	Y	K	20%
aa61–80	D	Y	V	F	I	Q	W	T	G	G	N	I	R	D	D	D	K	Y	T	H	33%
aa71–90	N	I	R	D	D	D	K	Y	T	H	F	F	S	G	F	C	N	T	M	C	20%
aa101–120	H	L	A	L	W	D	S	K	F	F	T	E	L	E	N	K	N	V	E	Y	27%
aa111–130	T	E	L	E	N	K	N	V	E	Y	V	V	I	I	E	N	D	N	V	I	27%
aa211–230	G	F	Y	F	E	I	A	R	I	E	N	E	M	K	I	N	R	Q	I	M	40%
aa221–240	N	E	M	K	I	N	R	Q	I	M	D	N	S	A	K	Y	V	E	H	D	20%
aa251–270	T	M	K	P	N	F	W	S	R	I	G	T	V	A	A	K	R	Y	P	G	47%
aa271–290	V	M	Y	T	F	T	T	P	L	I	S	F	F	G	L	F	D	I	N	V	20%
A35R-derived peptides (≥20% responders)																					
aa11–30	T	S	V	F	S	A	T	V	Y	G	D	K	I	Q	G	K	N	K	R	K	20%
aa21–40	C	T	V	P	T	M	N	N	A	K	L	T	S	T	E	T	S	F	N	D	27%
aa101–120	N	G	L	Y	Y	Q	G	S	C	Y	I	L	H	S	D	Y	K	S	F	E	20%
aa131–150	S	T	L	P	N	K	S	D	V	L	T	T	W	L	I	D	Y	V	E	D	27%
aa141–160	T	T	W	L	I	D	Y	V	E	D	T	W	G	S	D	G	N	P	I	T	27%
B6R-derived peptides (≥20% responders)																					
aa71–90	P	C	K	K	M	C	T	V	S	D	Y	V	S	E	L	Y	D	K	P	L	20%
aa91–110	Y	E	V	N	S	T	M	T	L	S	C	N	G	E	T	K	Y	F	R	C	20%

MVA-BN vaccinees (*n* = 7)	Peptide sequence																				RF
H3L-derived peptides (≥20% responders)																					
aa1–20	M	A	A	V	K	T	P	V	I	V	V	P	V	I	D	R	P	P	S	E	29%
aa11–30	V	P	V	I	D	R	P	P	S	E	T	F	P	N	V	H	E	H	I	N	29%
aa21–40	T	F	P	N	V	H	E	H	I	N	D	Q	K	F	D	D	V	K	D	N	29%
aa31–50	D	Q	K	F	D	D	V	K	D	N	E	V	M	Q	E	K	R	D	V	V	29%
aa61–80	D	Y	V	F	I	Q	W	T	G	G	N	I	R	D	D	D	K	Y	T	H	29%
aa71–90	N	I	R	D	D	D	K	Y	T	H	F	F	S	G	F	C	N	T	M	C	43%
aa161–180	K	V	K	T	E	L	V	I	D	K	D	H	A	I	F	T	Y	T	G	G	29%
aa171–190	D	H	A	I	F	T	Y	T	G	G	Y	D	V	S	L	S	A	Y	I	I	29%
aa181–200	Y	D	V	S	L	S	A	Y	I	I	R	V	T	T	A	L	N	I	V	D	43%
aa191–210	R	V	T	T	A	L	N	I	V	D	E	I	I	K	S	G	G	L	S	S	29%
aa201–220	E	I	I	K	S	G	G	L	S	S	G	F	Y	F	E	I	A	R	I	E	29%
aa221–240	N	E	M	K	I	N	R	Q	I	M	D	N	S	A	K	Y	V	E	H	D	57%
aa231–250	D	N	S	A	K	Y	V	E	H	D	P	R	L	V	A	E	H	R	F	E	29%
aa251–270	T	M	K	P	N	F	W	S	R	I	G	T	V	A	A	K	R	Y	P	G	29%
aa261–280	G	T	V	A	A	K	R	Y	P	G	V	M	Y	T	F	T	T	P	L	I	29%
aa271–290	V	M	Y	T	F	T	T	P	L	I	S	F	F	G	L	F	D	I	N	V	29%
A35R-derived peptides (≥20% responders)																					
aa21–40	C	T	V	P	T	M	N	N	A	K	L	T	S	T	E	T	S	F	N	D	29%

Response frequencies (RFs) of CD4⁺ T-cell responses to peptides from the MPXV proteins H3L, A35R, and B6R detected in at least 20% of individuals are shown for all mpox and MVA-BN participants combined (*n* = 22), individuals with mpox (*n* = 15), and MVA-BN vaccinees (*n* = 7). For each peptide, the position within the protein, the amino acid sequence, and RF are indicated.

**Table 3:**
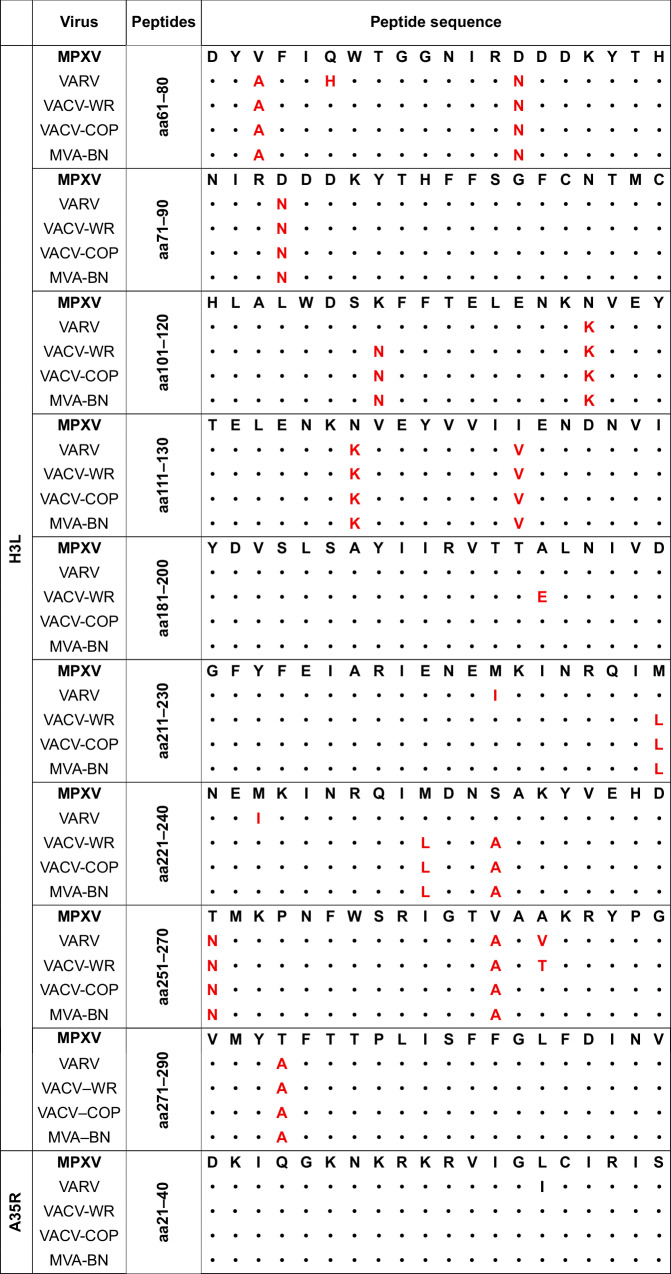
Amino acid sequences of the most frequently detected MPXV-derived peptides and comparison with the sequences of related Orthopoxviruses

Shown are MPXV-corresponding peptides that elicited virus-specific CD4^+^ T-cell responses in at least 20% of all analyzed individuals. The MPXV peptide sequences used for in vitro stimulation and ICS verification are listed, followed by the corresponding orthologous sequences from other Orthopoxviruses, including variola virus (VARV), vaccinia virus strain Copenhagen (VACV-COP), vaccinia virus strain Western Reserve (VACV-WR), and modified vaccinia virus Ankara Bavarian Nordic (MVA-BN). Amino acid residues differing from the MPXV-derived peptide sequences are highlighted in red and annotated accordingly.

### Immunogenicity of the H3L region aa251–270 is likely restricted by HLA-DRB1*01:01

**Fig. 3:**
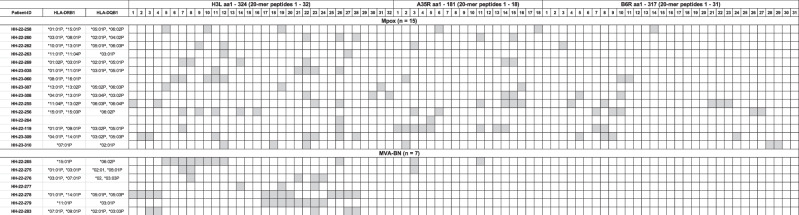
Peptide-level mapping of MPXV-specific CD4⁺ T-cell responses in mpox and MVA-BN–vaccinated individuals. Detailed map of MPXV-specific CD4⁺ T-cell responses to individual overlapping 20-mer peptides (10 amino acid overlap) spanning the MPXV glycoproteins H3L (324 amino acids, 32 peptides), A35R (181 amino acids, 18 peptides), and B6R (317 amino acids, 31 peptides). Each column represents an individual study participant, and each row corresponds to a single peptide. Gray-shaded boxes indicate positive responses detected by ICS. The left panel displays data from mpox individuals (*n* = 15), while the right panel shows MVA-BN–vaccinated donors (*n* = 7). HLA class II phenotypes (HLA-DRB1 and HLA-DQB1) are indicated for each donor. HLA typing data were available for 14 mpox and 6 MVA-BN participants; one donor from each group lacked HLA typing.

We performed in vitro binding assays on several HLA class II molecules (DRB1 and DQB1) to assess HLA restriction. High-affinity pairs were defined as those with an IC₅₀ ≤ 1000 nM, and very high-affinity pairs as those with an IC₅₀ ≤ 100 nM (Table 4). A peptide was considered likely HLA-restricted if at least 50% of participants carrying a particular HLA allele responded. Single-peptide responses per patient with known HLA-DRB1/-DQB1 or HLA-A/-B alleles to MPXV glycoproteins H3L, A35R, and B6R are shown in Fig. 3 for MPXV-specific CD4^+^ T-cell responses and in Supplementary Fig. 6 for MPXV-specific CD8^+^ T-cell responses, respectively.

**Table 4:** In vitro HLA-binding affinities of selected MPXV-derived peptides across multiple HLA-DRB1 and HLA-DQB1 molecules

Peptides		HLA-binding capacity	DQA1*01:01/DQB1*05:01	DQA1*01:02/DQB1*06:02	DQA1*03:01/DQB1*03:02	DQA1*05:01/DQB1*02:01	DQA1*05:01/DQB1*03:01	DRB1*01:01	DRB1*03:01	DRB1*04:01	DRB1*04:05	DRB1*07:01	DRB1*08:02	DRB1*09:01	DRB1*11:01	DRB1*12:01	DRB1*13:02	DRB1*15:01	Total bound (≤1000 nM)	Total bound (≤100 nM)
H3L	aa181–200	In vitro (IC50 nM)	429	648	279	38	153	26	441	295	220	0.2	10,248	4.4	435	60	32	6.0	15	7
		In silico (rank)	8	12	25	34	31	20	42	22	13	4.6	13	25	35	18	11	6.0		
		Responding patients	2/7	1/3	1/3	0/6	1/3	2/5	0/4	0/2	0/0	0/3	0/0	0/2	1/3	0/0	1/2	1/3		
	aa211–230	In vitro (IC50 nM)	54	12	136	30	698	292	101	85	24	99	681	19	284	17,950	11,252	63	14	8
		In silico (rank)	12	39	1.7	7.2	62	41	4.2	12	3.1	36	24	19	11	27	38	34		
		Responding patients	2/7	0/3	2/3	3/6	2/3	1/5	2/4	2/2	0/0	1/3	0/0	0/2	2/3	0/0	0/2	0/3		
	aa251–270	In vitro (IC50 nM)	15,838	822	1627	271	866	8.9	4965	13	65	33	87	224	34	65	2980	175	12	7
		In silico (rank)	63	8.8	48	58	13	4.2	47	0.84	3	12	0.83	11	6	11	19	27		
		Responding patients	3/7	1/3	1/3	1/6	1/3	3/5	1/4	1/2	0/0	0/3	0/0	1/2	1/3	0/0	1/2	1/3		
	aa91–110	In vitro (IC50 nM)	7.1	377	3102	13	22,738	865	3261	5265	132	11	203	35	6.5	588	50	3.1	12	7
		In silico (rank)	9.9	25	49	40	66	43	50	40	13	20	20	23	19	12	20	8.6		
		Responding patients	1/7	2/3	0/3	0/6	0/3	1/5	0/4	0/2	0/0	0/3	0/0	0/2	0/3	0/0	0/2	2/3		
	aa261–280	In vitro (IC50 nM)	31	2143	3019	62	2938	696	1628	396	51	0.93	1143	8.3	71	287	107	5.7	11	7
		In silico (rank)	19	65	46	29	41	38	69	38	15	2.5	46	14	48	27	29	8		
		Responding patients	1/7	0/3	0/3	1/6	1/3	1/5	0/4	0/2	0/0	1/3	0/0	1/2	1/3	0/0	0/2	0/3		
	aa201–220	In vitro (IC50 nM)	14	766	235	146	38	465	2790	1977	656	26	19,376	20	256	1237	2786	34	11	5
		In silico (rank)	2.5	35	11	13	7.7	42	65	44	19	28	54	23	56	47	34	34		
		Responding patients	1/7	0/3	2/3	1/6	2/3	1/5	1/4	0/2	0/0	1/3	0/0	0/2	2/3	0/0	0/2	0/3		
	aa101–120	In vitro (IC50 nM)	89	4453	38	122	–	31	755	389	54	21	–	180	845	1997	2977	7.5	11	6
		In silico (rank)	14	87	44	32	94	17	51	34	22	50	65	50	54	45	65	35		
		Responding patients	1/7	2/3	1/3	0/6	1/3	1/5	0/4	1/2	0/0	0/3	0/0	0/2	1/3	0/0	0/2	2/3		

**Peptides**		**HLA-binding capacity**	DQA1*01:01/DQB1*05:01	DQA1*01:02/DQB1*06:02	DQA1*03:01/DQB1*03:02	DQA1*05:01/DQB1*02:01	DQA1*05:01/DQB1*03:01	DRB1*01:01	DRB1*03:01	DRB1*04:01	DRB1*04:05	DRB1*07:01	DRB1*08:02	DRB1*09:01	DRB1*11:01	DRB1*12:01	DRB1*13:02	DRB1*15:01	**Total bound (≤1000 nM)**	**Total bound (≤100 nM)**
H3L	aa151–170	In vitro (IC50 nM)	1766	493	2905	831	3500	363	359	1248	1185	122	2819	622	1061	11,366	1414	167	7	0
		In silico (rank)	82	47	75	81	48	45	23	51	60	38	17	47	16	45	14	38		
		Responding patients	0/7	1/3	0/3	0/6	0/3	0/5	0/4	0/2	0/0	0/3	0/0	0/2	0/3	0/0	0/2	0/3		
H3L	aa81–100	In vitro (IC50 nM)	64	9201	22,585	–	9700	2966	–	135	704	603	–	781	5411	–	–	469	6	1
		In silico (rank)	65	83	58	64	67	85	88	73	63	84	91	88	84	90	94	81		
		Responding patients	1/7	2/3	0/3	0/6	0/3	0/5	0/4	0/2	0/0	0/3	0/0	0/2	0/3	0/0	0/2	2/3		
A35R	aa21–40	In vitro (IC50 nM)	4607	99	6352	6941	9092	1347	–	324	482	353	3734	4325	6610	4785	6218	313	5	1
		In silico (rank)	62	19	83	85	55	31	45	49	47	22	24	39	17	17	35	28		
		Responding patients	3/7	2/3	1/3	1/6	0/3	2/5	1/4	0/2	0/0	0/3	0/0	1/2	0/3	0/0	0/2	2/3		
H3L	aa41–60	In vitro (IC50 nM)	4985	–	1217	56	–	–	319	3559	324	–	–	–	–	–	–	3838	3	1
		In silico (rank)	27	84	13	9.7	94	94	4.7	54	29	91	84	95	88	69	40	84		
		Responding patients	1/7	2/3	0/3	0/6	0/3	1/5	0/4	0/2	0/0	0/3	0/0	0/2	0/3	0/0	1/2	2/3		
																		**Total**	**169**	**72**

Shown are HLA-binding affinities for MPXV-derived 20-mer peptides with 10-amino-acid overlap tested against 5 HLA-DQB1 and 11 HLA-DRB1 alleles. Binding affinity is expressed as the IC_50_ value (nM) of each peptide to its respective HLA molecule. High binding affinity was defined as IC_50_ < 1000 nM, and very high binding affinity as IC_50_ < 100 nM. The number of peptides binding to each HLA allele corresponds to the count of peptides below these thresholds, respectively. In silico binding predictions were obtained using the NetMHCIIpan 4.1 BA tool (IEDB Analysis Resource, version 2023.09), and results are reported as percentile ranks. The table also indicates the number of study participants carrying each specific HLA allele and the subset of these individuals who mounted a CD4^+^ T-cell response. A dash (–) indicates IC50 >30,000 nM, NetMHCIIpan 4.1 BA (Binding predictor-202309), data extracted on August 20, 2025, Germany DKMS—German Donors (*n* = 3,456,066), http://www.allelefrequencies.net.

Overall, we identified 169 high-affinity and 72 very high-affinity binding pairs. The peptides H3L_181-200, H3L_211-230, and H3L_251-270 demonstrated the broadest HLA-binding promiscuity, binding to 12–15 different HLA alleles. H3L_251-270 was likely restricted by DRB1*01:01 (IC_50_ = 8.9 nM; 3/5 responders). Additionally, H3L_211-230 was associated with DRB1*04:01 (IC_50_ = 85 nM; 2/2 responders), and A35R_21-40 with DQB1*06:02 (IC_50_ = 99 nM; 2/3 responders). Interestingly, we also observed several cases of frequent T-cell responses despite low in vitro binding affinities or vice versa. For example, 2 out of 3 DRB1*11:01 carriers responded to H3L_221-240, despite a high IC_50_ value of 6800 nM.

In conclusion, our findings show that peptides from the MPXV proteins H3L and A35R strongly bind to several DRB1 and DQB1 alleles. Notably, H3L_251–270 and H3L_211–230 are probably restricted by DRB1*01:01 and DRB1*04:01, respectively. Both of these correspond to the most frequently detected peptides in mpox patients.

### Reported OPXV T-cell epitopes differ only minimally from their MPXV counterparts at the amino acid level

**Fig. 4:**
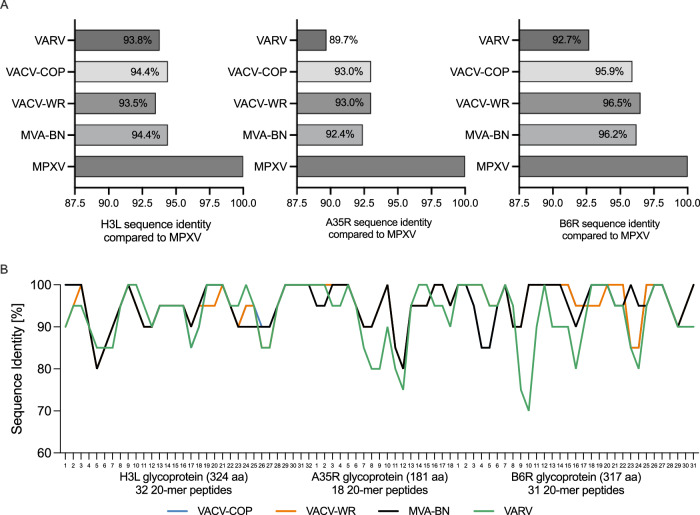
Sequence identity of MPXV glycoproteins H3L, A35R, and B6R across orthopoxviruses. **A** Protein sequence identity of MPXV Clade IIb A glycoproteins H3L (left), A35R (middle), and B6R (right) compared with their orthologs in Variola virus (VARV), Vaccinia virus strain Copenhagen (VACV-COP), Vaccinia virus strain Western Reserve (VACV-WR), and Modified Vaccinia Ankara virus (MVA-BN). Bars indicate the percentage of sequence identity relative to MPXV Clade IIb A. **B** Peptide-level comparison showing sequence identity for the experimentally tested overlapping 20-mer peptides (with 10 amino acid overlaps) derived from H3L, A35R, and B6R. Each data point represents the percentage of identical residues in the corresponding peptide relative to MPXV Clade IIb A. Colors indicate viral strains: VARV (green), MVA-BN (black), VACV-COP (blue), and VACV-WR (orange).

Next, we compared the sequence identity of the three full-length glycoproteins H3L, A35R, and B6R (Fig. 4A) and their 20-mer derivatives used in this study (Fig. 4B) between MPXV and other orthopoxviruses. The highest sequence identity between MPXV and MVA-BN was observed for B6R (96.5%), followed by H3L (94.4%) and A35R (92.4%). Notably, the MPXV sequences used here are identical to the lineage Clade IIb B.1 strain responsible for the 2022 mpox pandemic^30^. Additionally, we analyzed reported H3L, A35R, and B6R epitopes in MPXV and other orthopoxviruses using the Immune Epitope Database (IEDB.org), defining an epitope as non-homologous if its sequence differs by at least one amino acid. We identified previously reported T-cell epitopes in B6R (*n* = 27), H3L (*n* = 19), and A35R (*n* = 17). All these T-cell epitopes had been described in VACV (VACV-WR, -COP) or its derivatives (MVA) and in Cowpox virus (CPXV). Most listed epitopes were MHC class II-restricted (53–78%) and reported to be human-associated (53–78%). Sequence identities between reported MHC II-restricted T-cell epitopes and their orthologous MPXV sequences differed in 90% of cases for H3L, 45% for A35R, and 57% for B6R. Supplementary Table 3 summarizes the analyzed epitope data for H3L, A35R, and B6R.

### Immunogenetic imprint profiling of mpox resolvers and MVA-BN vaccinees

Finally, we examined the peripheral T-cell receptor (TCR) landscape for mpox-related signatures in six mpox and two MVA-BN individuals who had detectable CD4⁺ and/or CD8⁺ T-cell responses and enough material for sequencing. Because of the small sample size, all eight patient samples were combined and analyzed as a single MPOX discovery cohort.

**Fig. 5:**
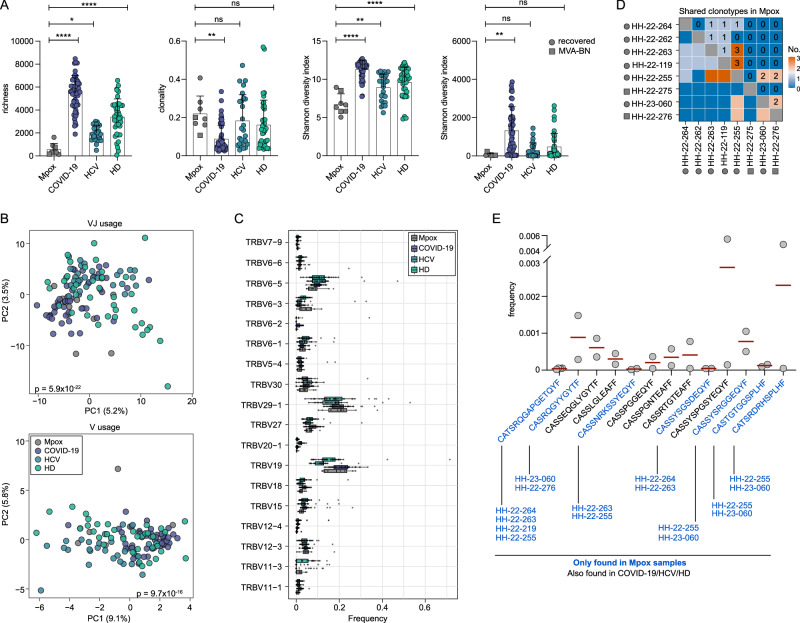
T-cell receptor (TCR) repertoire sequencing in individuals with mpox and MVA-BN vaccinees. **A** T-cell repertoire richness, clonality, and diversity measures in mpox individuals (*n* = 6) and MVA-BN vaccinated subjects (*n* = 2), collectively referred to as the MPOX cohort (dots represent mpox cases, squares represent MVA-BN vaccinees). Metrics from recovered COVID-19 patients (*n* = 50), hepatitis C virus (HCV)-infected individuals with sustained virological response after direct-acting antiviral treatment (SVR; *n* = 26), and healthy donors (HD; *n* = 38) served as control cohorts. **B** Principal component analysis (PCA) of TRBV and TRBV-J gene usage across Mpox, COVID-19, HCV, and HD cohorts. **C** Median frequency of TRBV gene usage within selected V gene families. **D** Heat map depicting inter- and intra-individual clonal overlap of unique TCR CDR3 clonotypes in the Mpox cohort, with mpox patients indicated by dots and MVA-BN vaccinated individuals by squares. **E** Mean frequencies of the 13 shared CDR3 clonotypes in the Mpox cohort. Clonotypes exclusively found in Mpox but absent in other cohorts are highlighted in blue.

Quantification of global repertoire metrics showed that MPOX samples had significantly reduced richness, especially compared to the COVID-19 cohort, where recently recovered individuals displayed notable T-cell expansion (Fig. 5A). MPOX samples also demonstrated lower diversity, although clonality was similar to HD and HCV controls, and no major differences were observed between mpox and MVA-BN vaccinees. Principal component analysis (PCA) of overall V and VJ gene usage did not reveal major group-specific repertoire skewing, although recovered COVID-19 individuals appeared to cluster more tightly within the V gene space (Fig. 5B). Overall, V-gene frequencies were mostly similar across groups, with MPOX samples showing a slight increase in TRBV19, TRBV6-3, TRBV29-1, and TRBV11-3 usage (Fig. 5C).

To analyze MPXV-specific T-cell signatures in more detail, we searched for shared clonotypes within the mpox cohort and identified 13 CDR3 sequences (Fig. 5D and Table 5). The most commonly used V genes were TRBV19 (*n* = 4; 31% of shared clonotypes), TRBV6-5 (*n* = 3; 23%), and TRBV15 (*n* = 2; 15%). All detected clonotypes had low frequencies (Fig. 5E). Seven clonotypes were exclusive to MPOX samples, while the remaining six were also found in at least one of the control groups, indicating their public nature. The most notable MPOX-exclusive clonotype (TRBV15/TRBD1/TRBJ2-5; CATSRQGAPGETQYF) was detected in 4 of 8 samples, with no clear HLA class I or II pattern for the sequenced alleles (HLA-A, -B, -DRB1, -DQB1). Importantly, we did not find this clonotype in major TCR databases (VDJdb, IEDB), further supporting its MPOX specificity.

**Table 5:**
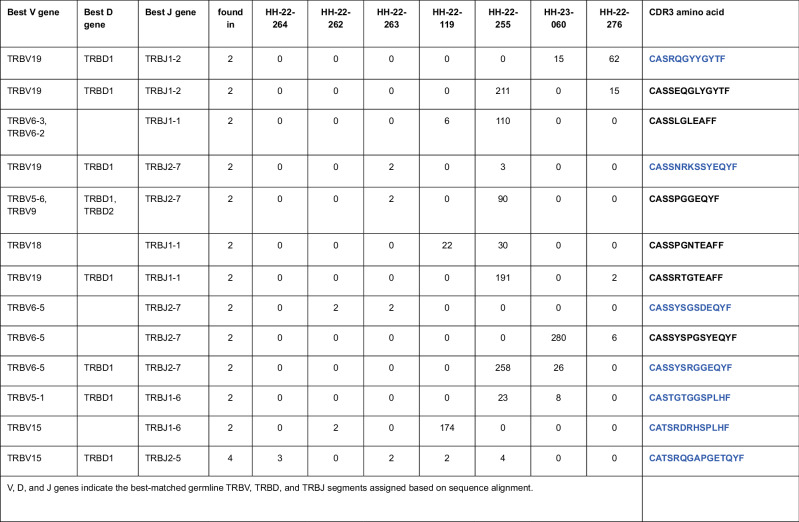
Shared TCRβ clonotypes identified in mpox

Thirteen shared clonotypes were identified among six mpox and two MVA-BN individuals with detectable MPXV-specific CD4⁺ and/or CD8⁺ T-cell responses and sufficient material for sequencing (no shared CDR3 was found for HH-22-275). Seven clonotypes were mpox-exclusive (blue).

To validate these findings, we sampled a small additional cohort of mpox recoverees (*n* = 7; validation cohort shown in Supplementary Fig. 7). Notably, this TRBV15 clonotype was again found in 3 of 7 individuals, confirming its association with MPOX infection.

## Discussion

This detailed immunological study presents the first comprehensive single-peptide-level analysis of MPXV-specific CD4^+^ and CD8^+^ T-cell responses to the glycoproteins H3L, A35R, and B6R, comparing immune responses between individuals with resolved mpox and those vaccinated with MVA-BN. Our systematic epitope characterization, using 81 overlapping 20-mer peptides and deconvolution after short-term MPXV peptide-specific in vitro cultivation, identified a broad range of low-level MPXV-specific epitopes and revealed distinct recognition patterns. These findings improve our understanding of MPXV cellular immunity and provide essential insights for developing next-generation vaccines.

Our results highlight the critical role of H3L as an immunogenic target, with all individuals in the mpox and MVA-BN groups mounting at least one response. This aligns with H3L being the main attachment protein of IMV and its high conservation among orthopoxviruses^31–33^. The broad range of H3L-specific CD4^+^ T-cell responses supports previous research and underscores its key role in both natural and vaccine-induced immunity^34,35^.

Notably, our epitope-level analysis revealed distinct recognition patterns between exposure groups. Individuals in the mpox group mostly recognized the peptides H3L_251–270 and H3L_211–230. In contrast, MVA-BN vaccinees most often targeted H3L_221–240. This variation suggests that small sequence differences between MPXV and MVA-BN might influence epitope recognition, despite an overall sequence identity of 94.4%, consistent with recent studies showing that even a single amino acid substitution can significantly alter T-cell recognition patterns^32^.

While H3L-specific CD4^+^ and CD8^+^ T-cell responses were consistent across participants, reactivity to A35R and B6R varied significantly in this small study. We observed less frequent A35R-specific and no B6R-specific CD4^+^ T-cell responses among MVA-BN vaccine recipients. These findings might be due to attenuating mutations introduced during MVA development^12^. Recent studies indicate that mRNA vaccine platforms targeting A35R and B6R are likely to induce stronger antibody responses than MVA-based vaccines and suggest that platform-specific limitations could explain these differences^33,36–38^.

A key part of our study was including six mpox patients who also received the MVA-BN vaccine, forming a hybrid-immunity group. Despite the added complexity, this provided valuable insights into immune-boosting effects. We observed a positive correlation between antigen exposure and the breadth of MPXV-specific CD4^+^ T-cell responses, suggesting that sequential exposure enhances cellular immunity. This aligns with studies showing hybrid immunity produces the most robust and long-lasting immunity to orthopoxviruses^28,39,40^. MVA-BN vaccinees with prior smallpox vaccination also demonstrate stronger, more durable antibody responses than naïve vaccinees, supporting heterologous prime-boost strategies^16,41^. Our data suggest this principle applies to MPXV-specific T-cell immunity, although further research is needed to fully understand hybrid immunity patterns and protection markers.

Our HLA-binding analysis revealed broad restriction patterns, supporting population-wide coverage. We identified high-affinity HLA-peptide binding pairs across DRB1 and DQB1 molecules, with H3L_251–270 binding strongly to DRB1*01:01 and H3L_211–230 likely restricted by DRB1*04:01. This HLA promiscuity, combined with high responses to H3L epitopes, reinforces their potential for universal vaccine inclusion. These findings confirm the translational potential of our experimentally defined epitopes^31^. Further studies will be essential for validating these interactions.

Finally, our correlation analyses showed temporal differences in epitope-specific T cell memory. We observed a negative correlation between time since mpox diagnosis and the breadth of virus-specific CD4^+^ responses to B6R, as well as CD8^+^ responses to both H3L and B6R. These findings indicate that B6R-specific T cell responses decline more rapidly than H3L-specific responses, which may explain why B6R-specific responses are absent in MVA-BN-vaccinated individuals, since MVA-BN antibody responses decrease over time, while responses from natural mpox infection tend to be more stable^42^. Notably, mpox reinfections have been reported, suggesting that neither vaccination, natural MPXV infection, nor hybrid immunity provides sterile immunity^43,44^. The distinct kinetics of epitope-specific responses have important implications for vaccine design and booster strategies. Long-term studies with extended follow-up are necessary to evaluate the durability of protective T-cell memory and determine optimal timing for boosters.

In line with the large viral genome and the wide range of possible target epitopes, our TCR repertoire analysis showed no major structural changes after mpox infection. However, we identified a TRBV15-encoded clonotype shared by about half of mpox patients, yet it was absent from other viral infection groups and not present in public TCR databases. Given the high immunogenicity of H3L, it is tempting to speculate that this clonotype might recognize an H3L-derived T-cell epitope within a specific HLA context. This hypothesis needs further validation, including identifying the paired TRα chain.

Our single-peptide T cell mapping resolves inconsistencies in cross-reactivity research. We found that 90% of previously identified VACV H3L MHC class II restricted T-cell epitopes have amino acid variations compared to MPXV sequences. Although in silico analyses showed 70% conservation, our data indicate that functional cross-reactivity might be lower due to differences at critical TCR contact residues^11^, which has significant implications for cross-protection and vaccine development.

Our experimental data on a small subset of HIV-positive individuals with good immune status who showed MPXV-specific T-cell responses similar to HIV-negative controls support the findings that antiretroviral therapy preserves pathogen-specific immunity^45,46^. This emphasizes the need for further research on MPXV-specific T-cell responses in at-risk populations, including HIV-infected individuals and those with low CD4^+^ T cell counts, to ensure broad protection.

Several limitations of this real-world immunological study must be acknowledged. First, because the MPXV peptide-specific T-cell responses were small, PBMCs had to be expanded in vitro. Furthermore, although our 20-mer peptide approach allowed for nearly comprehensive virus-specific CD4^+^ T-cell epitope mapping, it inherently introduced a bias against detecting optimal CD8^+^ T-cell responses^47^. Importantly, the statistical power for definitive between-group comparisons was limited by the relatively small cohort size available for further analysis, particularly in the MVA-BN group (*n* = 7), where only fresh PBMCs were used. Additionally, the hybrid immunity subgroup (six mpox cases with subsequent MVA-BN vaccination) complicated clear comparisons between natural and vaccine-induced immunity but offered valuable insights into boosting effects. The broad time frames, with mpox participants studied on average 338 days post-infection and MVA-BN vaccine recipients 272.5 days after their last immunization, introduced heterogeneity that affected direct comparisons and limited any conclusive interpretation regarding the breadth and durability of hybrid immunity or the MPXV-specific T-cell response. Finally, the exclusively male study population, though representative of the MPXV outbreak in Germany^48^, limits the generalizability concerning potential sex-based differences in immune responses^49,50^.

Therefore, key questions for future research include: (1) long-term memory durability, requiring longitudinal studies beyond 12 months to define the kinetics of epitope-specific maintenance; (2) protective correlates, establishing links between T-cell responses and clinical outcomes; (3) comprehensive T-cell epitope mapping, utilizing complementary technologies such as tetramer staining and single-cell approaches; (4) hybrid immunity, systematically examining how sequential infection and vaccination refine immune responses; (5) platform enhancement, developing next-generation vaccines that target H3L, A35R, and B6R more effectively; and (6) expanded protein analysis, characterizing the T-cell response to additional MPXV proteins using similar methodologies.

In conclusion, this study offers the first single-peptide-level mapping of MPXV T-cell epitopes, employing systematic deconvolution to identify platform-specific epitope targeting. We found that MVA-BN vaccinees mainly recognize H3L_221–240, while natural infections mostly target H3L_251–270 and H3L_211–230. The study also shows distinct timing in epitope-specific memory and provides comprehensive HLA restriction data, crucial for population coverage in vaccine development. Additionally, we observed that sequential exposure boosts immunity in a hybrid-immunity group, and identified a new TRBV15-encoded clonotype in MPXV-infected individuals, indicating a unique mpox-specific TCR signature. The widespread recognition of H3L by T cells and its broad HLA coverage make it a promising candidate for MPXV vaccine platforms. These findings, which specify immunogenic epitopes with defined HLA restrictions, pave the way for multi-epitope vaccines that could target cellular immunity more effectively than whole-virus approaches. Recent advances in mRNA vaccines targeting multiple MPXV antigens support this strategy^17,36,37,51,52^. Overall, this research fills important gaps in MPXV immunity, offering a more detailed understanding of epitope-specific responses beyond broad cross-reactivity assessments.

## Methods

### Clinical cohort

All individuals were recruited at the University Medical Center Hamburg-Eppendorf between July 2023 and November 2024. The study included participants who had recovered from acute mpox (mpox, *n* = 15), MPXV-naive individuals who were fully vaccinated with the live-attenuated Modified Vaccinia Ankara–Bavarian Nordic (MVA-BN) vaccine (Bavarian Nordic, Denmark) (*n* = 7), as well as six unexposed participants with no clinical history of mpox or MVA-BN vaccination (*n* = 6). Among mpox individuals, six had received the MVA-BN vaccine before sample collection and thus were analyzed for the quality of their hybrid immunity. All study participants (*n* = 28), including individuals not exposed to MVA-BN or MPXV, were male.

The study was conducted in accordance with the Declaration of Helsinki and received formal approval from the local ethics committee of the Ärztekammer Hamburg (PV4780, 2022-100911-BO-ff). All participants were fully informed about the study’s goals and procedures and provided written informed consent prior to participation. For the mpox group, infection was confirmed by polymerase chain reaction (PCR) using skin-lesion swabs and/or serum samples. The HIV status of all participants, including MVA-BN-vaccinated individuals and unexposed controls, was determined by serology and RT-PCR. MVA-BN-vaccinated and unexposed individuals tested negative for MPXV by PCR at the time of analysis. HLA class I and II typing was performed at the Institute of Transfusion Medicine, University Medical Center Hamburg-Eppendorf, using PCR–sequence-specific oligonucleotide (SSO) technology (One Lambda, Canoga Park, CA, USA) on venous blood samples.

### Sample processing and MPXV peptide-specific T-cell expansion

PBMCs were isolated from venous blood and expanded as previously described^26,27^. Briefly, cells were cultured in RPMI 1640 medium (Thermo Fisher Scientific, Cat# 11875085) supplemented with 100 IU/mL penicillin-streptomycin (Thermo Fisher Scientific, Cat# 15140122), 1% HEPES buffer solution (Thermo Fisher Scientific, Cat# 15630080), and 10% fetal bovine serum (Sigma-Aldrich, Cat# F7524). For antigen-specific expansion, PBMCs were stimulated for 11–12 days with peptide pools (10 µg/mL per peptide, 150–180 µg/mL total peptide concentration) covering the full length of MPXV proteins H3L, A35R, and B6R. These pools consisted of 15–18 20-mer peptides with a 10-amino acid overlap, based on the MPXV-M5312_HM12_Rivers reference isolate (NCBI GenBank: MT903340.1; sequences in Supplementary Table 4). Co-stimulation was provided by adding 1 µL/mL of anti-CD28/anti-CD49d (BD Biosciences, Cat# 347690) at the start of the culture, whereas 50 IU/mL rIL-2 (Miltenyi Biotec, Cat# 130-097-743) was added initially and renewed every second day throughout the culture period. After expansion, MPXV peptide-specific T-cell responses were assessed via IFN-γ ELISpot as previously described^26,27^.

### IFN-γ ELISpot assay

**Fig. 6:**
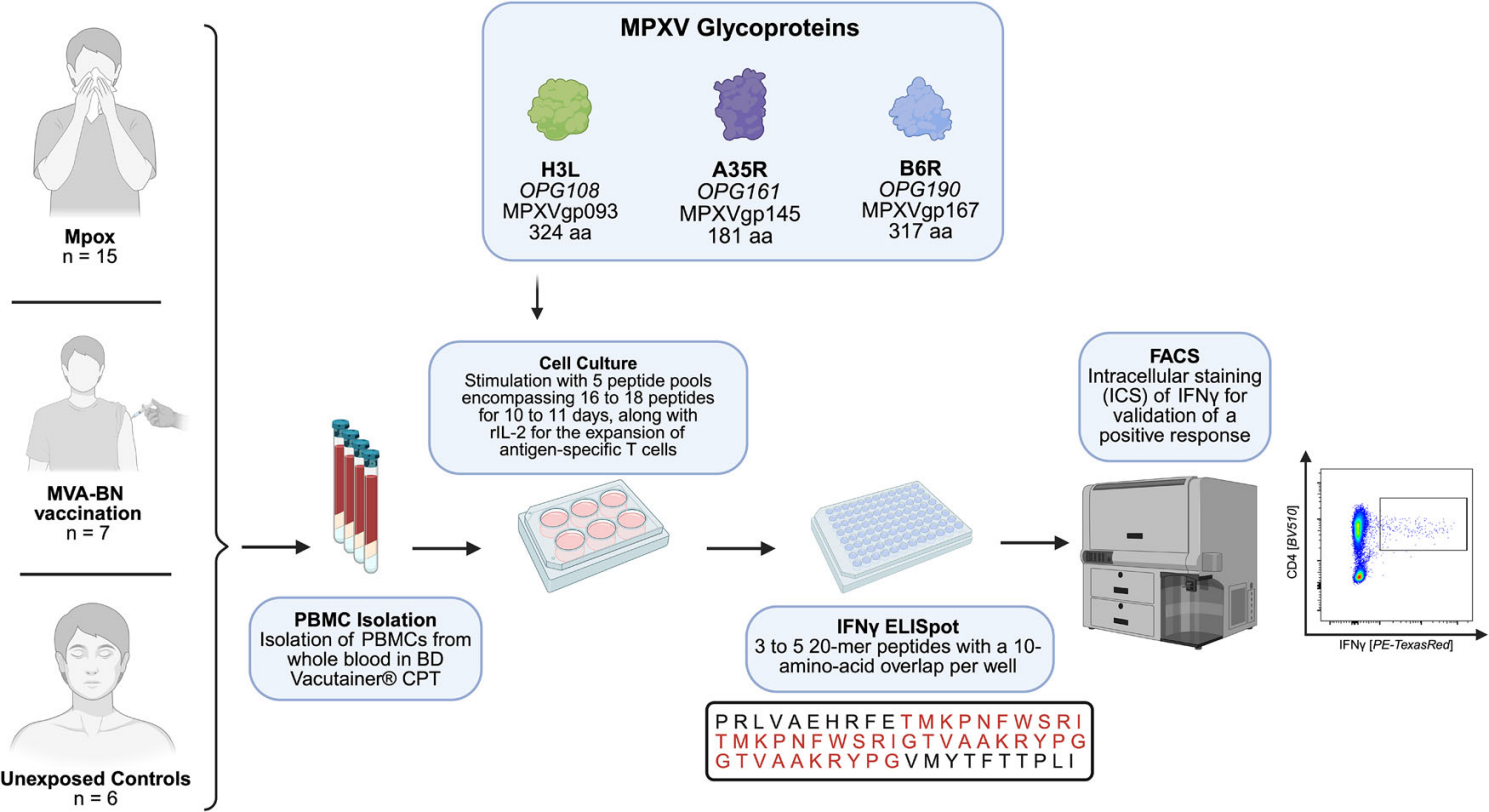
Experimental workflow for mapping MPXV-specific T-cell responses. PBMCs were collected from mpox individuals (*n* = 15), MVA-BN-vaccinated donors (*n* = 7), and unexposed controls (*n* = 6). After isolation using Vacutainer® CPT™ tubes, PBMCs were expanded for 10–11 days with IL-2 and anti-CD28/anti-CD49d in the presence of overlapping 20-mer peptide pools (10 amino acid overlap) covering the MPXV glycoproteins H3L, A35R, and B6R. Expanded T cells were re-stimulated with smaller peptide pools (3–5 peptides) in IFN-γ ELISpot assays. Responses exceeding twofold background were considered positive. Reactive peptides were further validated by ICS for IFN-γ in CD4⁺ T cells, with positivity defined as ≥3× the frequency observed in unstimulated controls.

A total of 100,000 cells per well were restimulated overnight with peptide pools containing 3–5 20-mer MPXV peptides (10 µg/mL for each peptide) on plates pre-coated with anti-IFN-γ (clone 1-D1K, Mabtech). Anti-CD3 and unstimulated cells served as controls. IFN-γ secretion was detected using biotinylated anti-IFN-γ (clone 7-B6-1, Mabtech), streptavidin-ALP, and BCIP/NBT substrate (all Mabtech) according to the manufacturer’s protocol. An ELISpot well was considered positive if the spot count was at least two times higher than the negative control. Positive responses were further analyzed by flow cytometry to identify CD4^+^ and CD8^+^ T-cells. The workflow is shown in Fig. 6.

### Intracellular cytokine staining

Positive ELISpot responses were confirmed by intracellular cytokine staining (ICS). Cells were restimulated with individual peptides (10 µg/mL, 16 h) at 37 °C, with 5% CO₂. Brefeldin A (5 µg/mL, Sigma-Aldrich) was added after 1 h. Cells were stained with LIVE/DEAD Near-IR (BioLegend) and surface antibodies: anti-CD3 (UCHT1, AF700), anti-CD4 (SK3, BV510), anti-CD8 (RPA-T8, PerCP-Cy5.5), anti-CD14 (63D3, APC-Cy7), and anti-CD19 (HIB19, APC-Cy7; all BioLegend).

Following fixation/permeabilization (FoxP3 Staining Buffer Set, eBioscience), intracellular IFN-γ was stained (4S.B3, PE-Dazzle594; BioLegend). Data were acquired on a BD FACSymphony A3 using FACSDiva v8. Responses were considered positive if IFN-γ^+^ frequencies were ≥3-fold above negative controls and ≥0.025% of the CD4^+^ and CD8^+^ parent population, respectively. The gating strategy is shown in Supplementary Fig. 8.

### In vitro and in silico HLA binding prediction

In vitro HLA binding assays were conducted using purified HLA class II molecules, specifically DRB1 and DQB1, and 18 MPXV peptides from H3L, A35R, and B6R. An HLA molecule was considered to bind the respective peptide with high affinity if the IC50 value was equal to or less than 1000 nM. Additionally, in silico binding predictions were obtained using the NetMHCIIpan 4.1 BA (IEDB Analysis Resource, version 2023.09) HLA II prediction tool. Results are presented as a peptide’s percentile rank for a specific HLA molecule, with low percentile values indicating a high affinity.

### T-cell receptor sequencing

Genomic DNA was isolated from cryopreserved PBMCs using the DNeasy Blood & Tissue Kit (Qiagen, Hilden, Germany). The VDJ-rearranged T-cell receptor beta chain (TRB) loci were amplified using a multiplex PCR and the BIOMED-2 TRB-B primer pool, as previously described^53^. Amplicons were quality-controlled on an Agilent TapeStation and pooled to a final concentration of 8 nM. Sequencing and demultiplexing were performed on the Illumina MiSeq platform with v3 chemistry (Illumina, San Diego, CA) and paired-end reads (2 × 300 bp) at an average coverage of 80,000 reads per sample. The rearranged TRB loci were aligned using MiXCR (v3.0.12)^54^ and its default reference genome library. Only productive TCR rearrangements with at least two read counts were considered for downstream analyses. Each unique CDR3 nucleotide sequence was defined as a clonotype. For metric calculation and the V/VJ architecture, repertoires were proportionally normalized to 50,000 read counts. As reference groups, we included healthy donors (HD; *n* = 38; median age 32, range 19–61, 58% female; Supplementary Table 5), individuals recovered from mild/moderate COVID-19 during the first pandemic wave (*n* = 50; median age 40.5, range 8–70, 52% female; Supplementary Table 6)^55^, and HCV-infected individuals with sustained virological response (SVR) after direct-acting antiviral therapy (DAA) (*n* = 26; median age 55, range 32–78, 54% female; Supplementary Table 7)^56^.

### TCR repertoire analyses

We calculated TCR richness, clonality, and diversity as non-redundant and complementary metrics describing the clonal architecture of T-cell repertoires. While TCR richness indicates the total number of unique clonotypes, clonality quantifies the degree of potential virus-specific clonotype expansion or serves as an indicator of an oligoclonal repertoire with limited diversity. Clonality was calculated as 1 − Pielou’s evenness index^57^, which measures clonotype dominance within the repertoire (a value of 0 denotes maximal clonal diversity, whereas a value of 1 indicates the predominance of a single clonotype). To assess TCR diversity, we computed the Shannon entropy, which integrates both clonotype richness and relative clonal frequencies (evenness) using the formula: ${\rm{H}}1=-{\sum }_{i=1}^{N}{P}_{i}\log \left({P}_{i}\right)$, where pi represents the proportional frequency of each clonotype. In addition, the inverse Simpson index (1/*D*), with $D={\sum }_{i=1}^{R}{p}_{i}^{2}$, where *R* is the richness and *p*_*i*_ the proportional abundances. While both metrics describe repertoire diversity, they differ in sensitivity. The Shannon entropy emphasizes the contribution of rare clonotypes. It thus captures overall richness and evenness, whereas the inverse Simpson index is more influenced by highly abundant clonotypes and places greater weight on dominant expansions. Clonal overlap and VDJ architecture were assessed using the tcR (v2.3.2) and immunarch (v0.9.1) packages. All analyses and data visualization were performed using R (v4.3.3; R Core Team, 2024) or GraphPad Prism (version 10).

### Data analysis and statistics

Flow cytometric data were analyzed using FlowJo version 10 for Windows (TreeStar, Ashland, OR, USA). Statistical analyses and graph generation were performed with GraphPad Prism version 10.3.0 for Mac (GraphPad Software, San Diego, CA, USA). Comparisons of T-cell responses between mpox and MVA-BN individuals, as well as between HIV-positive and HIV-negative individuals, were conducted using the Mann–Whitney *U* test. The Wilcoxon signed-rank test was applied for paired comparisons between the three MPXV proteins and between CD4⁺ and CD8⁺ T-cell responses. Correlations between variables were assessed using Spearman’s rank correlation coefficient. All tests were two-tailed, and *p* values ≤ 0.05 were considered statistically significant. To compare immune metrics, one-way ANOVA with Tukey’s multiple comparisons test was used. Group differences in VDJ gene usage were assessed using principal component analysis (PCA), and the statistical significance of multivariate separation was tested with a MANOVA based on Pillai’s trace after confirming variance homogeneity with Bartlett’s test. Statistical significance levels are indicated as follows: *p < 0.05; **p < 0.01; ***p < 0.001; ****p < 0.0001.

## Supplementary information

Supplementary information

## Data Availability

The immune repertoire data from mpox patients are available at ENA (Project: PRJEB112266). Other datasets generated and/or analyzed during the current study are not publicly available due to patient confidentiality and data protection regulations but are available from the corresponding author on reasonable request.

## Code availability

Peptide binding predictions were performed using the publicly available software NetMHCIIpan 4.1. No custom code was generated for this study.
