# Supplementary material for: MPXV H3L elicits broadly directed CD4 T cell responses in mpox patients and MVA vaccinees
Source: Npj Viruses. 2026 Jul 7;4:31. doi: 10.1038/s44298-026-00205-5 (PMC13342585; doi:10.1038/s44298-026-00205-5)
Supplement: Supplementary file 1 — Supplementary information [file 44298_2026_205_MOESM1_ESM.pdf]

Supplementary Figure 1. MPXV peptide-specific CD4⁺ and CD8⁺ T cell responses in mpox without MVA-BN vaccination, hybrid mpox individuals with MVA-BN vaccination and MVA-BN vaccinated subjects only.

Comparison of $CD4^+$ T cell responses among mpox individuals without MVA-BN vaccination (n = 9), hybrid individuals mpox followed by MVA-BN vaccination (n = 6), and MVA-BN–vaccinated donors (n = 7).

(A) Breadth of H3L-, A35R- and B6R-specific IFN-γ^+ $CD4^+$ T cell responses, defined as the number of peptide-specific responses per individual.

(B) Magnitude of H3L-, A35R- and B6R-specific IFN-γ^+ $CD4^+$ T cell responses, represented by the average frequency of IFN-γ -producing $CD4^+$ T cells within the total $CD4^+$ T cell population.

(C) Breadth of H3L-, A35R- and B6R-specific IFN-γ^+ $CD8^+$ T cell responses.

(D) Magnitude of H3L-, A35R- and B6R-specific IFN-γ^+ $CD8^+$ T cell responses.

All panels show responses against overlapping 20-mer peptides (10 amino acid overlap) derived from the MPXV glycoproteins H3L, A35R, and B6R. Statistical analyses were performed using the Mann–Whitney U test; significance is indicated as *$p < 0.05$, **$p < 0.01$, ***$p < 0.001$, and ns = not significant.

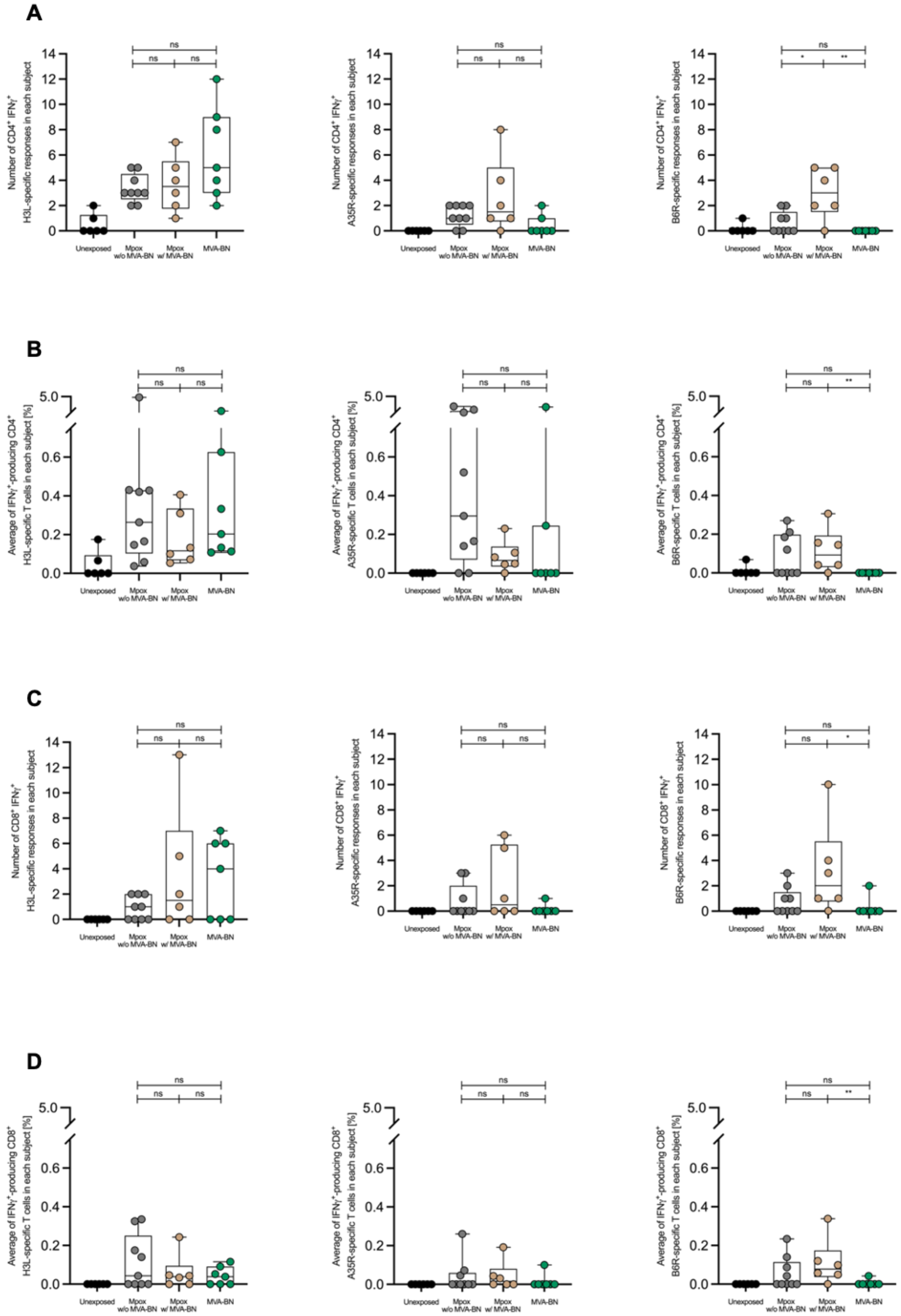
A
Number of CD4+ IFNγ+ H3L-specific responses in each subject
Number of CD4+ IFNγ+ A35R-specific responses in each subject
Number of CD4+ IFNγ+ B6R-specific responses in each subject
B
Average of IFNγ+-producing CD4+ H3L-specific T cells in each subject [%]
Average of IFNγ+-producing CD4+ A35R-specific T cells in each subject [%]
Average of IFNγ+-producing CD4+ B6R-specific T cells in each subject [%]
C
Number of CD8+ IFNγ+ H3L-specific responses in each subject
Number of CD8+ IFNγ+ A35R-specific responses in each subject
Number of CD8+ IFNγ+ B6R-specific responses in each subject
D
Average of IFNγ+-producing CD8+ H3L-specific T cells in each subject [%]
Average of IFNγ+-producing CD8+ A35R-specific T cells in each subject [%]
Average of IFNγ+-producing CD8+ B6R-specific T cells in each subject [%]
Unexposed
Mpox w/o MVA-BN
Mpox w/ MVA-BN
MVA-BN
ns
*
**

Supplementary Figure 2. Comparison of MPXV-specific T-cell responses in mpox individuals stratified by HIV status.

Analysis of H3L-, A35R- and B6R-specific IFN-γ^+ $CD4^+$ and $CD8^+$ T-cell responses in mpox individuals (n = 15), stratified into HIV-negative (n = 10) and HIV-positive (n = 5) groups.

(A) Breadth of H3L-, A35R- and B6R-specific IFN-γ^+ $CD4^+$ T cell responses, defined as the number of peptide-specific responses per individual.

(B) Magnitude of H3L-, A35R- and B6R-specific IFN-γ^+ $CD4^+$ T cell responses, represented by the average frequency of IFN-γ -producing $CD4^+$ T cells within the total $CD4^+$ T cell population.

(C) Breadth of IFN-γ^+ $CD8^+$ T-cell responses.

(D) Magnitude of IFN-γ^+ $CD8^+$ T cell responses.

All panels are based on intracellular cytokine staining following stimulation with overlapping 20-mer peptides (10 amino acid overlap) spanning MPXV glycoproteins H3L, A35R, and B6R. No significant differences were observed between HIV-negative and HIV-positive individuals (Mann–Whitney U test, ns = not significant).

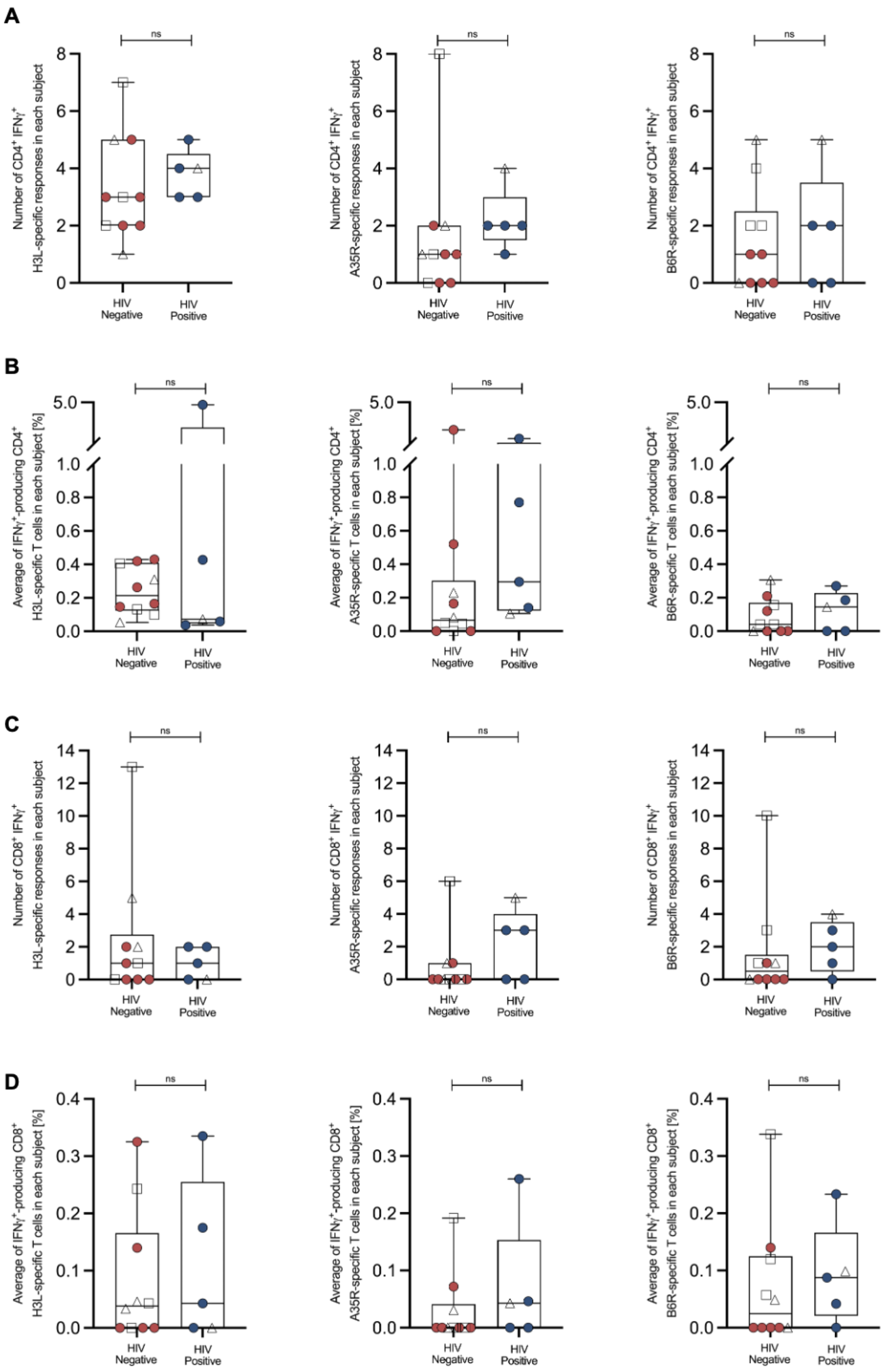

A
B
C
D
ns
Number of CD4+ IFNγ+ H3L-specific responses in each subject
Number of CD4+ IFNγ+ A35R-specific responses in each subject
Number of CD4+ IFNγ+ B6R-specific responses in each subject
Average of IFNγ+-producing CD4+ H3L-specific T cells in each subject [%]
Average of IFNγ+-producing CD4+ A35R-specific T cells in each subject [%]
Average of IFNγ+-producing CD4+ B6R-specific T cells in each subject [%]
Number of CD8+ IFNγ+ H3L-specific responses in each subject
Number of CD8+ IFNγ+ A35R-specific responses in each subject
Number of CD8+ IFNγ+ B6R-specific responses in each subject
Average of IFNγ+-producing CD8+ H3L-specific T cells in each subject [%]
Average of IFNγ+-producing CD8+ A35R-specific T cells in each subject [%]
Average of IFNγ+-producing CD8+ B6R-specific T cells in each subject [%]
HIV Negative
HIV Positive

Supplementary Figure 3. Comparison of $CD4^+$ and $CD8^+$ T-cell responses to MPXV glycoproteins H3L, A35R, and B6R.

Comparison of IFN-γ^+ $CD4^+$ and $CD8^+$ T-cell responses in mpox ($n = 15$) and MVA-BN–vaccinated individuals ($n = 7$).

(A) Breadth (number of peptide-specific responses) of $CD4^+$ and $CD8^+$ T cells targeting H3L (left), A35R (middle), and B6R (right) in mpox participants.

(B) Magnitude (average frequency of IFN-γ -producing $CD4^+$ or $CD8^+$ T cells) for the same proteins in mpox participants.

(C) Breadth (left) and magnitude (right) of H3L-specific responses in MVA-BN–vaccinated donors. Responses were determined by intracellular cytokine staining after stimulation with overlapping 20-mer peptides (10-aa overlap) spanning each MPXV glycoprotein. Statistical analyses were performed using the Mann–Whitney U test; $p < 0.05$ (*), $p < 0.01$ (**), ns = not significant.

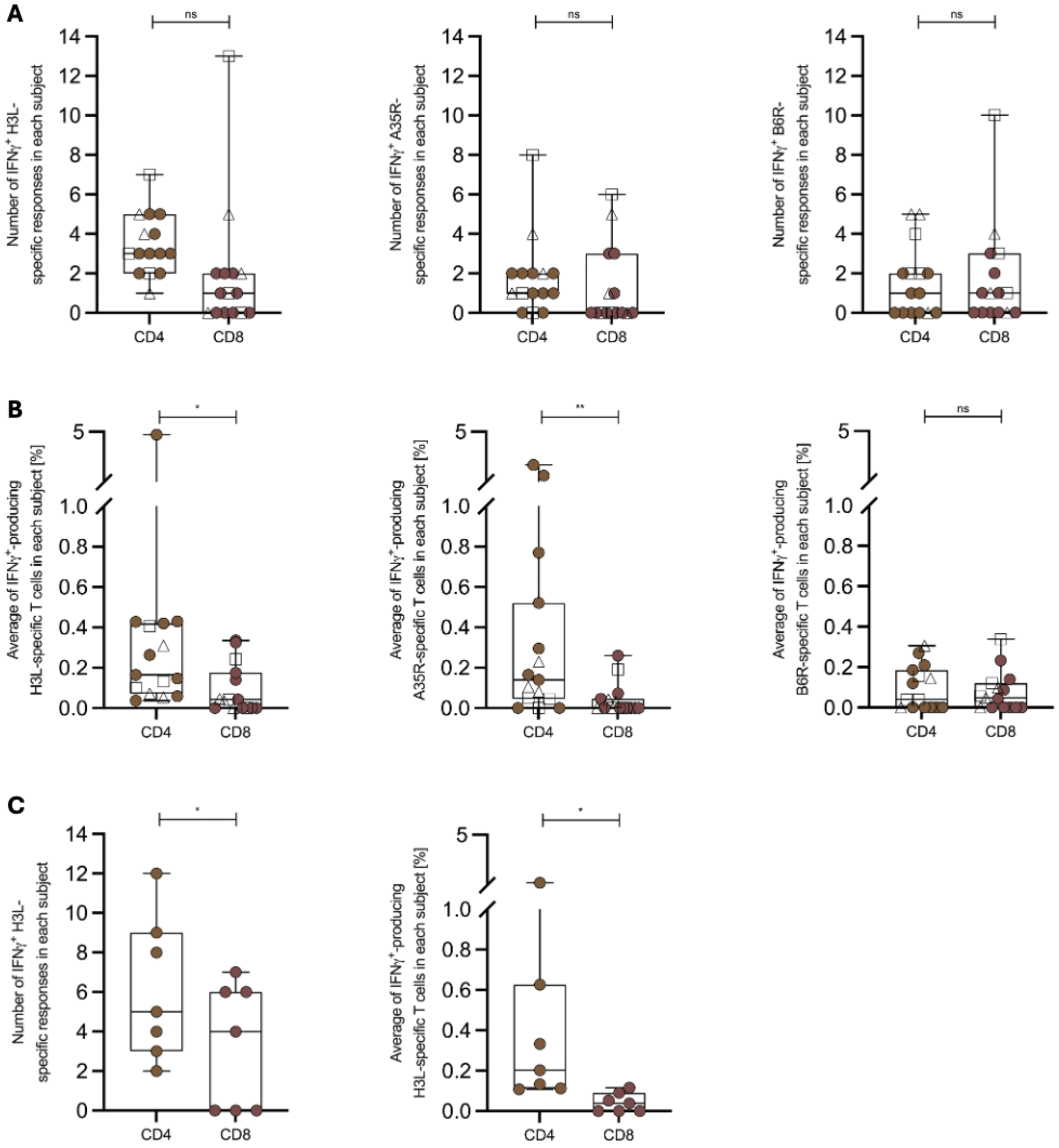
A
ns
Number of IFNγ+ H3L-specific responses in each subject
Number of IFNγ+ A35R-specific responses in each subject
Number of IFNγ+ B6R-specific responses in each subject
CD4
CD8
B
*
**
ns
Average of IFNγ+-producing H3L-specific T cells in each subject [%]
Average of IFNγ+-producing A35R-specific T cells in each subject [%]
Average of IFNγ+-producing B6R-specific T cells in each subject [%]
C
Number of IFNγ+ H3L-specific responses in each subject
Average of IFNγ+-producing H3L-specific T cells in each subject [%]

Supplementary Figure 4. MPXV peptide-specific CD8$^+$ T-cell responses to MPXV glycoproteins H3L, A35R, and B6R following infection and vaccination.

(A) Breadth of MPXV peptide-specific IFN-γ$^+$ CD8$^+$ T cell responses, represented as the number of reactive peptides per individual, for H3L (left), A35R (middle), and B6R (right) in unexposed controls (n = 6), mpox individuals (n = 15), and MVA-BN–vaccinated donors (n = 7). Hybrid individuals (mpox + MVA-BN) are indicated by triangles (single MVA-BN dose) and squares (two MVA-BN doses).

(B) Magnitude of MPXV peptide-specific IFN-γ$^+$ CD8$^+$ T cell responses, shown as the mean frequency of IFN-γ -producing CD8$^+$ T cells within the total CD8$^+$ T-cell population, for the same groups and glycoproteins as in (A).

(C) Distribution of MPXV peptide-specific IFN-γ$^+$ CD8$^+$ T cell responses across MPXV glycoproteins H3L (gray), A35R (yellow), and B6R (blue), expressed as the percentage of total peptide-specific responses within each group: mpox without MVA-BN vaccination (n = 9), MVA-BN (n = 7), and hybrid mpox with MVA-BN vaccination (n = 6).

(D) Spearman correlation matrix of immunological parameters in mpox individuals (n = 15), including age, time since infection, number of antigen exposures (1 = mpox only, 2 = mpox + 1× MVA-BN, 3 = mpox + 2× MVA-BN), and breadth and magnitude of CD4$^+$ and CD8$^+$ T-cell responses for each MPXV glycoprotein (H3L, A35R, and B6R). Color intensity represents the correlation coefficient (ρ), ranging from –1 (black) to +1 (white). Red asterisks (*) denote statistically significant correlations ($p < 0.05$). Correlations were calculated using two-tailed Spearman rank tests. Age data were unavailable for one participant.

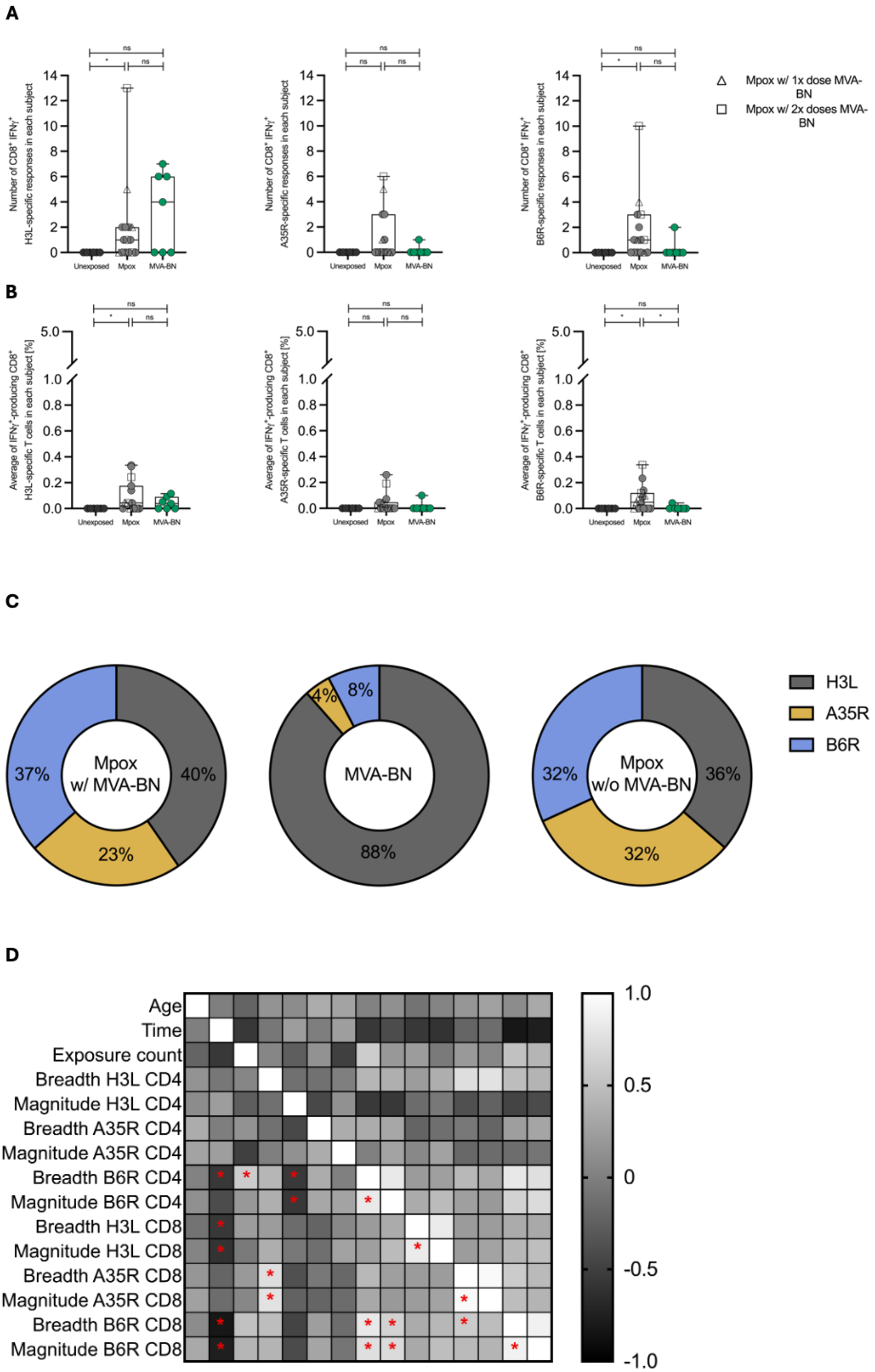

A
Number of CD8+ IFNγ+ H3L-specific responses in each subject
Number of CD8+ IFNγ+ A35R-specific responses in each subject
Number of CD8+ IFNγ+ B6R-specific responses in each subject
Unexposed
Mpox
MVA-BN
ns
*
Mpox w/ 1x dose MVA-BN
Mpox w/ 2x doses MVA-BN
B
Average of IFNγ+-producing CD8+ H3L-specific T cells in each subject [%]
Average of IFNγ+-producing CD8+ A35R-specific T cells in each subject [%]
Average of IFNγ+-producing CD8+ B6R-specific T cells in each subject [%]
C
Mpox w/ MVA-BN
40%
23%
37%
MVA-BN
88%
4%
8%
Mpox w/o MVA-BN
36%
32%
32%
H3L
A35R
B6R
D
Age
Time
Exposure count
Breadth H3L CD4
Magnitude H3L CD4
Breadth A35R CD4
Magnitude A35R CD4
Breadth B6R CD4
Magnitude B6R CD4
Breadth H3L CD8
Magnitude H3L CD8
Breadth A35R CD8
Magnitude A35R CD8
Breadth B6R CD8
Magnitude B6R CD8
1.0
0.5
0
-0.5
-1.0

Supplementary Figure 5. Mapping of MPXV-specific CD8$^+$ T-cell responses to overlapping peptides of H3L, A35R, and B6R.

(A) Frequency of MPXV-specific IFN-γ$^+$ CD8$^+$ T cell responses to each peptide among all analyzed individuals (n = 22; mpox group + MVA-BN vaccinated individuals).

(B) Response frequencies in only mpox individuals (n = 15).

(C) Response frequencies in MVA-BN–vaccinated donors only (n = 7).

Each bar represents the percentage of individuals exhibiting an MPXV peptide-specific IFN-γ$^+$ CD8$^+$ T cell response to a given 20-mer peptide (with 10 amino acid overlaps) derived from MPXV glycoproteins H3L (32 peptides), A35R (18 peptides), and B6R (31 peptides). Peptides recognized by ≥ 20% of subjects are labeled with their respective peptide number and amino acid range.

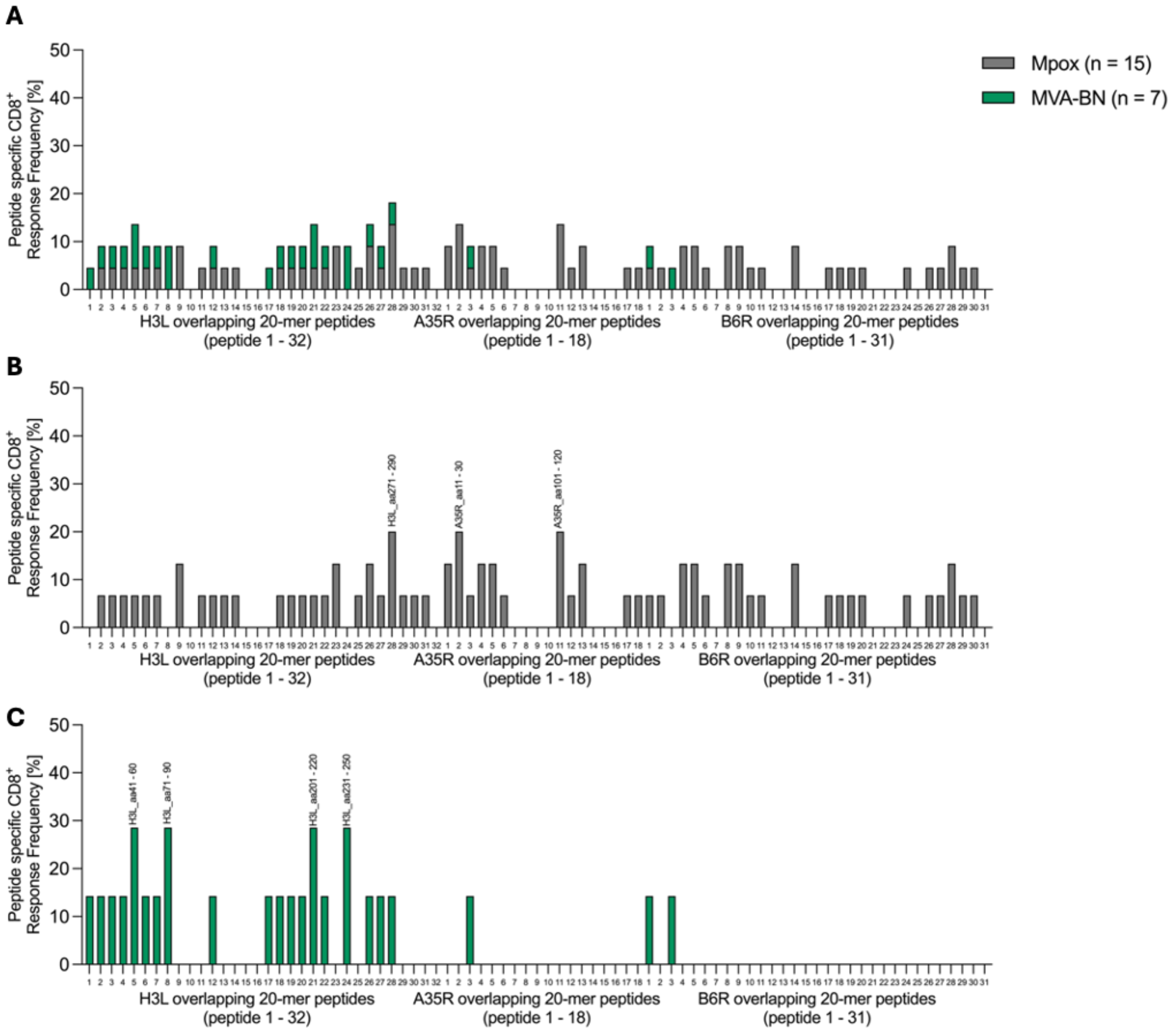
A
B
C
Peptide specific CD8+ Response Frequency [%]
Mpox (n = 15)
MVA-BN (n = 7)
H3L overlapping 20-mer peptides (peptide 1 - 32)
A35R overlapping 20-mer peptides (peptide 1 - 18)
B6R overlapping 20-mer peptides (peptide 1 - 31)
H3L_aa271 - 290
A35R_aa11 - 30
A35R_aa101 - 120
H3L_aa41 - 60
H3L_aa71 - 90
H3L_aa201 - 220
H3L_aa231 - 250

Supplementary Figure 6. Peptide-level mapping of MPXV-specific CD8$^+$ IFN-γ$^+$ T-cell responses in mpox and MVA-BN–vaccinated individuals.

Heatmap summarizing MPXV-specific IFN-γ$^+$ CD8$^+$ T cell responses to individual 20-mer peptides (10-aa overlap) spanning the MPXV glycoproteins H3L (324 aa, 32 peptides), A35R (181 aa, 18 peptides), and B6R (317 aa, 31 peptides). Each column represents an individual donor, and each row represents a single peptide. Gray-shaded boxes indicate positive responses in the intracellular cytokine staining (ICS) assay. The left panel shows mpox (n = 15), and the right panel shows MVA-BN–vaccinated donors (n = 7). HLA class I molecules (HLA-A and HLA-B) are indicated for each study participant. HLA data were available for 14 mpox and 6 MVA-BN participants; one individual per group lacked HLA typing.

			H3L aa1 - 324 (20-mer peptides 1 - 32)																															
Patient-ID	HLA-A	HLA-B	1	2	3	4	5	6	7	8	9	10	11	12	13	14	15	16	17	18	19	20	21	22	23	24	25	26	27	28	29	30	31	32
Mpox (n = 15)																																		
HH-22-258	*03:01P, *29:02P	*07:02P, *44:03P																														
*HH-22-260	*02, *03:01P	*08:01P, *44:02P																														
HH-22-262	*01:01P, *02:01P	*37:01P, *57:01P																														
HH-22-263	*02:01P, -	*35:02P, *44:02P																														
*HH-22-269	*24:02P, *30:01P	*08:01P, *14:02P																														
HH-23-035	*01:01P, *68:01P	*35:01P, *51:01P																														
*HH-23-060	'02:01P A*03:01F	27:02P B*35:01P																														
HH-23-307	*30:02P, *68:02P	*07:02P, *49:01P																														
*HH-23-308	*02:01P	*15:01P, *18:01P																														
*HH-22-255	*32:01P, *68:01P	*07:02P, *44:02P																														
HH-22-256	*25:01P, *68:02P	*18:01P, *44:03P																														
HH-22-264																																
HH-22-119	*30:01P, *31:01P	*13:02P, *44:02P																														
HH-23-309	*01:01P, *29	*07:02P, *44:04P																														
HH-23-310	*03:01P, *26:01P	*14:01P, *14:02P																														
MVA-BN (n = 7)																																		
HH-22-265	*02:01P, *11:01P	*07:02P, *15:01P																														
HH-22-275	*02:01P, *23:01P	*15:01P, *50:01P																														
HH-22-276	*02:01P, *68:03P	*18:01P, *57:01P																														
*HH-22-277																																
HH-22-278	*02:01P, *31:01P	*27:05P, *49:01P																														
HH-22-279	*24:02P, *68:01P	*40:02P, *44:02P																														
HH-22-283	*02:01P, -	'13:02P, *40:01P																														

	A35R aa1 - 181 (20-mer peptides 1 - 18)																	
Patient-ID	1	2	3	4	5	6	7	8	9	10	11	12	13	14	15	16	17	18
Mpox (n = 15)																		
HH-22-258																		
*HH-22-260																		
HH-22-262																		
HH-22-263																		
*HH-22-269																		
HH-23-035																		
*HH-23-060																		
HH-23-307																		
*HH-23-308																		
*HH-22-255																		
HH-22-256																		
HH-22-264																		
HH-22-119																		
HH-23-309																		
HH-23-310																		
MVA-BN (n = 7)																		
HH-22-265																		
HH-22-275																		
HH-22-276																		
*HH-22-277																		
HH-22-278																		
HH-22-279																		
HH-22-283																		

	B6R aa1 - 317 (20-mer peptides 1 - 31)																													
Patient-ID	1	2	3	4	5	6	7	8	9	10	11	12	13	14	15	16	17	18	19	20	21	22	23	24	25	26	27	28	29	30	31
Mpox (n = 15)																															
HH-22-258																															
*HH-22-260																															
HH-22-262																															
HH-22-263																															
*HH-22-269																															
HH-23-035																															
*HH-23-060																															
HH-23-307																															
*HH-23-308																															
*HH-22-255																															
HH-22-256																															
HH-22-264																															
HH-22-119																															
HH-23-309																															
HH-23-310																															
MVA-BN (n = 7)																															
HH-22-265																															
HH-22-275																															
HH-22-276																															
*HH-22-277																															
HH-22-278																															
HH-22-279																															
HH-22-283																															

Supplementary Figure 7. TCR immune repertoire sequencing of the mpox validation cohort.

(A) Mean metrics of the mpox validation cohort (n = 7) compared to the mpox discovery cohort shown in main Figure 6.

(B) PCA of VJ and V gene architecture.

(C) Median frequencies of selected TRBV genes.

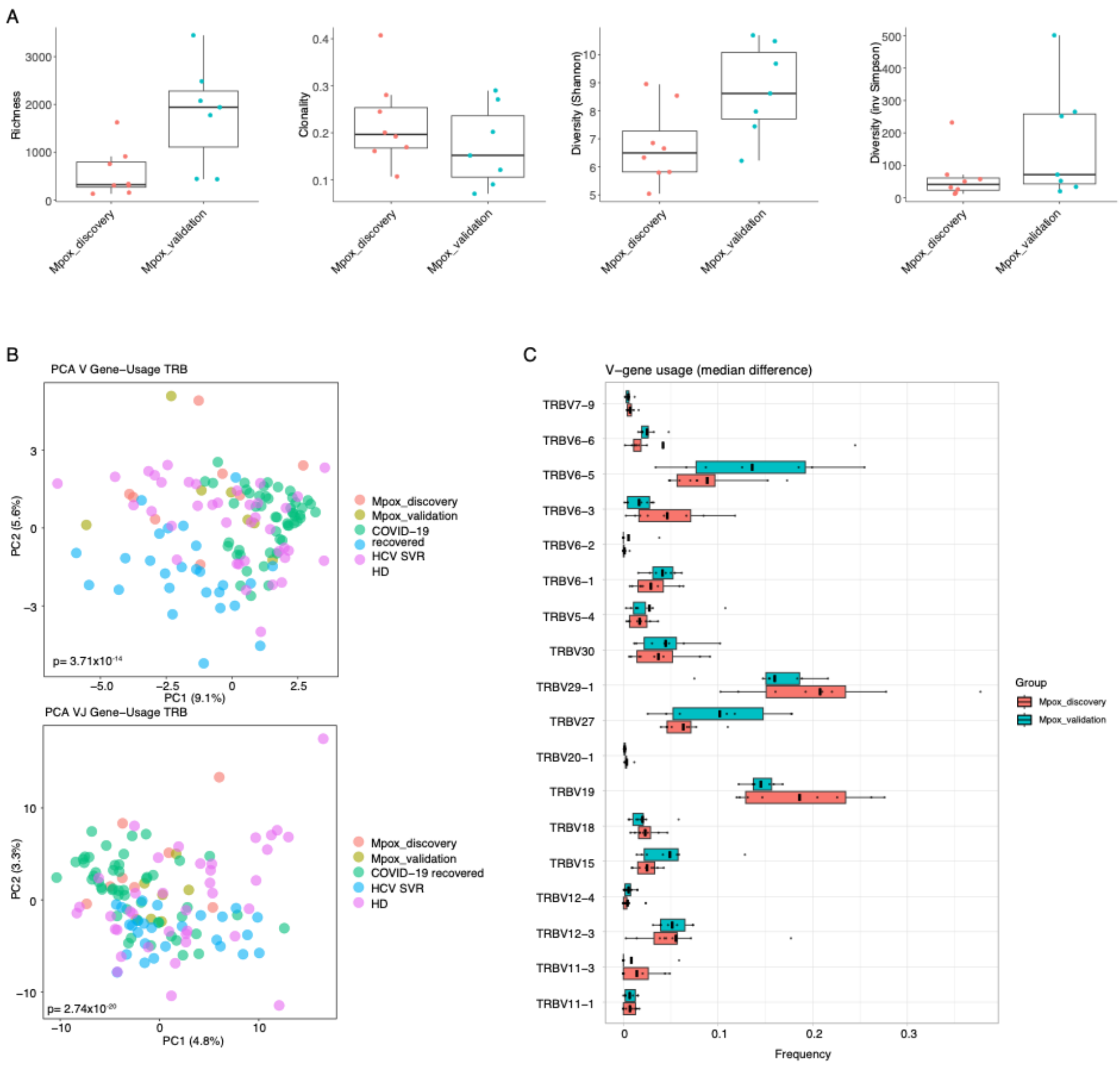

Supplementary Figure 8. Gating strategy for identification of MPXV-specific IFN-γ^{+} CD4^{+} and CD8^{+} T cells.

Flow cytometry gating strategy used to identify MPXV-specific IFN-γ -producing CD4^{+} and CD8^{+} T cells following peptide stimulation. Single-cell lymphocytes were gated based on forward and side scatter (FSC-A vs. SSC-A), followed by doublet exclusion (FSC-A vs. FSC-H). Live cells were selected by excluding dead cells stained with Live/Dead™ APC-Cy7 dye. T cells were gated as CD3^{+} (Alexa Fluor 700), and subsequently CD4^{+} and CD8^{+} subsets were defined using CD4 (BV510) and CD8 (PerCP-Cy5.5) markers. IFN-γ production was detected by intracellular staining using IFN-γ (PE-Dazzle 594).

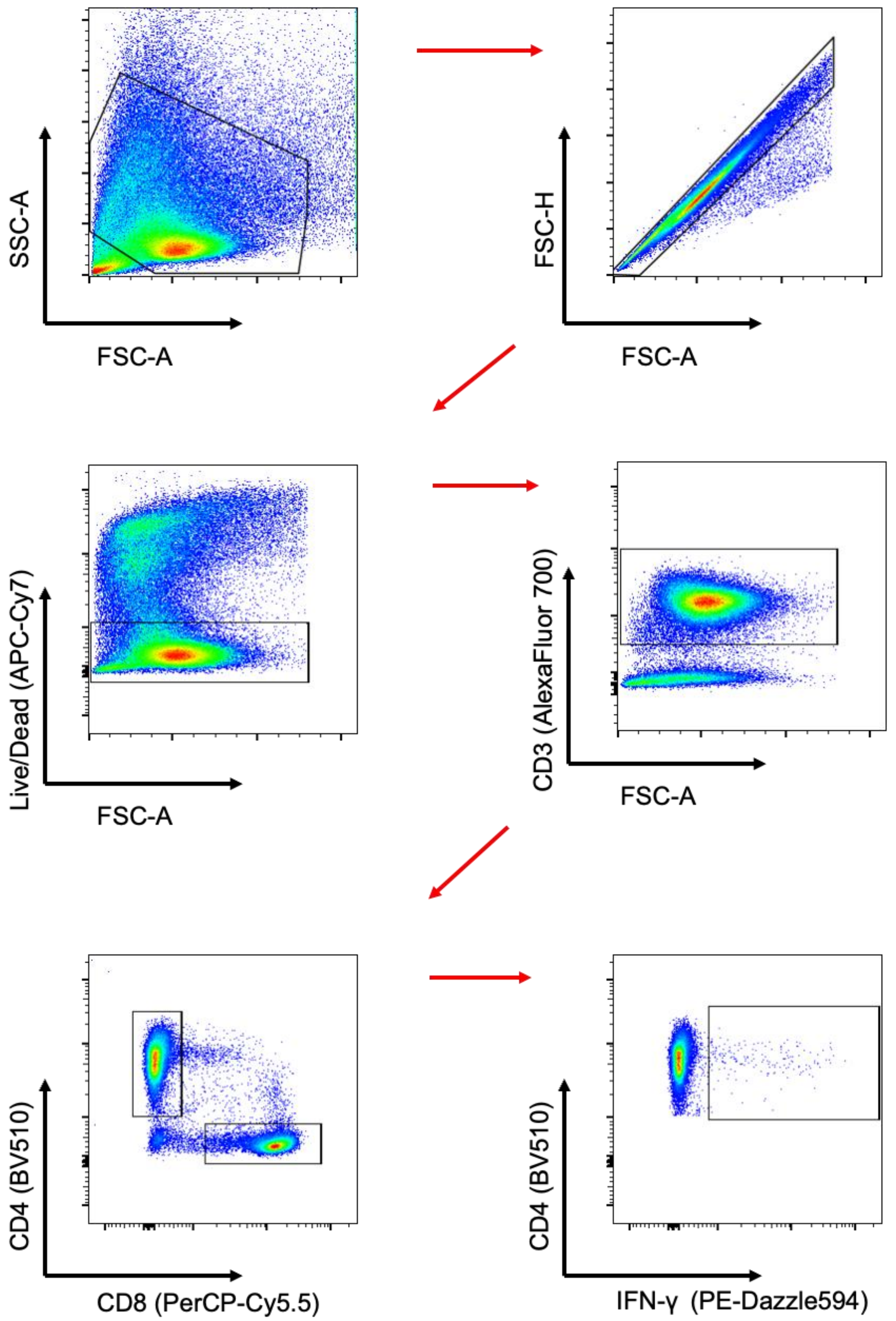

SSC-A
FSC-A
FSC-H
FSC-A
Live/Dead (APC-Cy7)
FSC-A
CD3 (AlexaFluor 700)
FSC-A
CD4 (BV510)
CD8 (PerCP-Cy5.5)
CD4 (BV510)
IFN-γ (PE-Dazzle594)

Supplementary Table 1. Clinical characteristics of individuals with acute or convalescent mpox infection.

Demographic and clinical data are shown for individuals with acute (n = 3) or convalescent mpox infection (n = 14).

Patient ID: Internally assigned pseudonym to ensure patient confidentiality and data anonymity.

Age/Sex: Age at the time of inclusion and biological sex (m = male, f = female).

MVA-BN: Vaccination status with the Modified Vaccinia Ankara - Bavarian Nordic vaccine.

Mpox diagnosis: Confirmation of infection via PCR.

Other diseases / Medication: Relevant comorbidities (e.g., HIV status, if applicable) and concurrent pharmacological treatments.

Lymphocytes per µL: Absolute lymphocyte count measured in peripheral blood.

Lymphocyte Subsets: Absolute counts (cells/µL) for total T cells ($CD3^+$), B cells ($CD19^+$), as well as $CD4^+$ (T helper cells) and $CD8^+$ (cytotoxic T cells) subpopulations were determined via flow cytometry.

Patient ID	Age	Sex	MVA-BN		Mpox diagnosis			Other diseases		Medication	Lymphocytes per µL				
			1st	2nd	1st	2nd	Fever	HIV	Other		Total	T	B	CD4+	CD8+
**HH-22-254**	34	m	27.07.22	07.11.22	27.07.22	no	yes	Neg			NA	NA	NA	NA	NA
**HH-22-255**	49	m	08.07.22	no	23.08.22	no	no	Pos	RA	MTX, Alendronat	1778	1405	135	747	587
**HH-22-256**	35	m	29.07.22	no	15.06.22	no	NA	Neg			NA	NA	NA	NA	NA
**HH-22-258**	49	m	no	no	19.07.22	no	yes	Neg			NA	NA	NA	NA	NA
**HH-22-257**	49	m	12.10.22	08.04.24	28.06.22	11.09.23	no	Neg			NA	NA	NA	NA	NA
**HH-22-259**	41	m	11.09.23	no	30.07.22	08.09.23	Yes	Neg	Asthma		NA	NA	NA	NA	NA
**HH-22-262**	39	m	no	no	17.06.22	no	yes	Neg			NA	NA	NA	NA	NA
**HH-22-263**	25	m	no	no	28.06.22	no	yes	Neg			NA	NA	NA	NA	NA
**HH-22-264**	42	m	11.07.22	no	15.08.22	no	no	Neg			NA	NA	NA	NA	NA
**HH-22-269**	41	m	no	no	13.07.22	no	NA	Pos			1847	1404	76	517	831
**HH-22-284**	46	m	08.08.22	10.11.22	15.03.24	no	NA	Neg			NA	NA	NA	NA	NA
**HH-22-285**	28	m	no	no	18.03.24	no	no	Neg			NA	NA	NA	NA	NA
**HH-22-260**	39	m	no	no	30.06.22	no	yes	Pos			2242	1726	211	695	1054
**HH-22-265**	NA	m	NA	NA	NA	NA	NA	NA			NA	NA	NA	NA	NA
**HH-23-308**	40	m	no	no	27.09.24	no	no	Pos			2999	2429	261	1529	840
**HH-23-309**	36	m	22.07.22	10.12.22	27.11.24	no	no	Neg			1973	NA	NA	NA	NA
**HH-23-310**	32	m	26.01.24	26.02.24	12.11.24	no	no	Neg			1384	NA	NA	NA	NA

Supplementary Table 2. CD8⁺ T-cell response frequencies to commonly detected MPXV-derived peptides.

Shown are the most frequently recognized peptides from the MPXV proteins H3L, A35R, and B6R that elicited CD8⁺ T-cell responses in at least 20% of individuals. Data are presented for all analyzed participants (n = 22), individuals with mpox (n = 15), and MVA-BN vaccinees (n = 7). The peptide amino acid sequences and their positions within the respective proteins are indicated.

**Mpox (n = 15)**	**Peptide sequence**	**RF**
H3L-derived peptides (≥20% responders)		
aa271–290	V M Y T F T T P L I S F F G L F D I N V	20%
A35R-derived peptides (≥20% responders)		
aa11–30	T S V F S A T V Y G D K I Q G K N K R K	20%
aa101–120	N G L Y Y Q G S C Y I L H S D Y K S F E	20%
**MVA-BN vaccinees (n = 7)**	**Peptide sequence**	**RF**
H3L-derived peptides (≥20% responders)		
aa41–60	E V M Q E K R D V V I V N D D P D H Y K	29%
aa71–90	N I R D D D K Y T H F F S G F C N T M C	29%
aa201–220	E I I K S G G L S S G F Y F E I A R I E	29%
aa231–250	D N S A K Y V E H D P R L V A E H R F E	29%

Supplementary Table 3. List of known epitopes for MPXV proteins H3L, A35R, and B6R.

This table compiles all previously reported epitopes for H3L, A35R, and B6R from the Immune Epitope Database (IEDB), including orthologous proteins from other orthopoxviruses as of September 15, 2025. For each epitope, the sequence, amino acid position within MPXV, and any amino acid differences from the reference MPXV sequence are listed. Epitopes are annotated as HLA-restricted, with the specific MHC allele responsible for recognition indicated.

Protein	Virus	Species	Epitope Sequence	Position	MPXV Sequence	Modifications (Ref. > MPXV)	MHC Restriction	Reference
H3L	VACV	H. sapiens	WDSNFFTELENKK	105–117	WDSKFFTELENKN	108 (N > K)<br>117 (K > N)	HLA class II	[1]
H3L	VACV	H. sapiens	FFTELENKKVEYV	109–121	FFTELENKNVEYV	117 (K > N)	HLA-DRB1*01:01	[1]
H3L	VACV	H. sapiens	VIEDITFLRPVLK	129–141	VIEDITFLRPVLK		HLA-DRB1*15:01, HLA-DRB5*01:01	[1]
H3L	VACV	H. sapiens	ITFLRPVLKAMHD	133–145	ITFLRPVLKAIHD	143 (M > I)	HLA-DRB1*15:01, HLA-DRB5*01:01	[1]

H3L	VACV	H. sapiens	ELVMDKN HAIFTY	165–177	ELVIDKD HAIFTY	168 (M > I) 171 (N > D) 173[2] (T > A)	HLA-DRB3*02:02	[1]
H3L	VACV VACV-WR	H. sapiens	NEMKINR QILDNA	221–233	NEMKINR QIMDNS	230 (L > M) 233 (A > S)	HLA class II, HLA-DRB1*04:01, HLA-DRB4*01:01, HLA-DRB4*01:01	[1,2]
H3L	VACV VACV-WR	H. sapiens	INRQILDN AAKYV	225–237	INRQIMD NSAKYV	230 (L > M) 233 (A > S)	HLA class II, HLA-DRB3*03:01	[1,2]
H3L	VACV NYCBH - Dryvax	H. sapiens	ILDNAAK YV	229–237	IMDNSAK YV	230 (L > M) 233 (A > S)	HLA-DRB4*01:01	[3]
H3L	VACV	H. sapiens	ILDNAAK YVEHDP	229–241	IMDNSAK YVEHDP	230 (L > M) 233 (A > S)	HLA-DRB3*03:01	[1]
H3L	VACV VACV-WR	M. musculus	PGVMYAF TTPLISFF	269–283	PGVMYTF TTPLISFF	274 (A > T)	H2-IAb, H2-b class II	[4-7]
A35R	VACV-COP	H. sapiens	TVYGDKI QGKNKR KRV	17–32	TVYGDKI QGKNKR KRV	20[3] (R > G)	HLA class II	[8]
A35R	VACV-COP	H. sapiens	RVIGLCIR ISMVISL	31–45	RVIGLCIR ISMVISL		HLA-DR1, HLA-DR3, HLA-DR4, HLA-DR7, HLA-DR15, HLA-DRB4	[9]
A35R	VACV-COP	H. sapiens	LSMITMS AFLIVRLN	46–60	LSMITMS AFLIVRLN		HLA-DR1, HLA-DR3, HLA-DR4, HLA-DR7, HLA-DR11, HLA-DR13, HLA-DR15, HLA-DRB3, HLA-DRB4, HLA-DRB5	[9]
A35R	VACV-COP	H. sapiens	SAFLIVRL NQCMSA N	52–66	SAFLIVRL NQCMSA N		HLA-DR1, HLA-DR3, HLA-DR4, HLA-DR7, HLA-DR11, HLA-DR13, HLA-DR15	[9]
A35R	VACV-COP	H. sapiens	AFLIVRLN QCMSAN EA	53–68	AFLIVRLN QCMSAN KA	67 (E > K)	HLA class II	[8]
A35R	VACV-COP	H. sapiens	SSTTQYD HKESCN GLY	89–104	SSTTQYD HKESCN GLY		HLA class II	[8]

A35R	VACV-COP	H. sapiens	NGLYYQGSCYILHSD	101–115	NGLYYQGSCYILHSD		HLA-DR1, HLA-DR3, HLA-DR13, HLA-DR15	[9]
A35R	VACV-COP	H. sapiens	CYILHSDYQLFSDAKA	109–124	CYILHSDYKSFEDAKA	117 (Q > K)<br>118 (L > S)<br>120 (S > E)	HLA class II	[8]
A35R	VACV-COP	H. sapiens	SDYQLFSDAKANCTA	114–128	SDYKSFEDAKANCAA	117 (Q > K)<br>118 (L > S)<br>120 (S > E)<br>127 (T > A)	HLA-DR3, HLA-DR4	[9]
A35R	VACV-WR	M. musculus	YQLFSDAKANCTAES	116–130	YKSFEDAKANCAAES	117 (Q > K)<br>118 (L > S)<br>120 (S > E)<br>127 (T > A)	H2-IAb	[5]
A35R	VACV NYCBH-Dryvax	H. sapiens	TKTTSDYQDSDVSQ	160–173	TKTTSDYQDSDVSQ		HLA-DQ	[3]
B6R	VACV-COP	H. sapiens	MKTISVVTLLCVLPA	1–15	MKTISVVTLLCVLPA		HLA class II	[9]
B6R	VACV-COP	H. sapiens	ISVVTLLCVLPAVVY	4–18	ISVVTLLCVLPAVVY		HLA-DR1, HLA-DR4, HLA-DR7, HLA-DR13, HLA-DR15, HLA-DRB3, HLA-DRB5	[9]
B6R	VACV-COP	H. sapiens	SVVTLLCVLPAVVYS	5–19	SVVTLLCVLPAVVYS		HLA class II	[10]
B6R	VACV-COP	H. sapiens	CTVPTMNNAKLTSTET	21–36	CTVPTMNNAKLTSTET		HLA class II	[8]
B6R	VACV-COP	M. musculus	AKLTSTETSFNNNQKV	29–44	AKLTSTETSFNDKQKV	40[2, 3](N > D)<br>41[2, 3](N > K)	H2-d class II	[8]
B6R	VACV-COP	M. musculus	FTCDQGYHSSDPNAV	46–60	FTCDSGYHSLDPNAV	50[2, 3] (Q > S)<br>55 (S > L)	H2-d class II	[5,11]
B6R	VACV-COP	M. musculus	PNAVCETDKWKYENPC	57–72	PNAVCETDKWKYENPC		H2-d class II	[8]
B6R	MVA	M. musculus	SDYISELYNKPLYEV	79–93	SDYVSELYDKPLYEV	82 (I > V)<br>87 (N > D)	H2-IAb	[9]
B6R	VACV-COP	H. sapiens	LYNKPLYEVNSTMTLS	85–100	LYDKPLYEVNSTMTLS	87 (N > D)	HLA-DR1, HLA-DR3, HLA-DR4, HLA-DR7, HLA-DR11, HLA-DR13, HLA-DR15,	[8]

							HLA-DRB4, HLA-DRB5	
B6R	VACV -COP	*H. sapiens*	TKYFRCE EKNGNTS W	105–119	TKYFRCE EKNGNTS W		HLA class II	[10]
B6R	VACV -COP	*H. sapiens*	EYMTINC DVG	151–160	EYMTINC DVG	153[3] (I > M)	HLA-DR1, HLA-DR3, HLA-DR4, HLA-DR11, HLA-DR13, HLA-DR1, HLA-DR3, HLA-DR4, HLA-DR11, HLA-DR13	[12]
B6R	VACV VACV -COP	*H. sapiens*	EYMTINC DVGYEVI GASYIS	151–170	EYMTINC DVGYEVI GVSYIS	153[3] (I > M) 166 (A > V)	HLA class II	[12]
B6R	VACV -COP	*H. sapiens*	ITINCDVG YEVIGAS Y	153–168	MTINCDV GYEVIGV SY	153[3] (I > M) 166 (A > V)	HLA-DR1, HLA-DR3, HLA-DR4, HLA-DR7, HLA-DR11, HLA-DR13, HLA-DR15, HLA-DRB3, HLA-DRB4	[8]
B6R	VACV -COP	*H. sapiens*	NSWNVIP SCQQKC DI	174–188	NSWNVIP SCQQKC DI	188[1] (M > I)	HLA-DR4, HLA-DR11	[9]
B6R	VACV -COP	*H. sapiens*	NGLISGS TFSIGGVI	193–207	NGLISGS TFSIGGVI		HLA class II	[10]
B6R	VACV -COP	*H. sapiens*	SGSTFSI GGVIHLS CK	197–212	SGSTFSI GGVIHLS CK		HLA-DR3, HLA-DR7, HLA-DR15, HLA-DR3, HLA-DR7, HLA-DR15	[8]
B6R	VACV -COP	*M. musculu s*	FSIGGVIH LSCKSGF I	201–216	FSIGGVIH LSCKSGF T	216[2, 3] (I > T)	H2-d class II	[8]
B6R	VACV -COP	*H. sapiens*	GGVIHLS CKSGFILT	204–218	GGVIHLS CKSGFTL T	216[2, 3] (I > T)	HLA-DR15	[9]
B6R	VACV -COP	*H. sapiens*	CIDGKWN PILPTCVR	225–239	CIDGKWN PILPTCVR	233[1, 2] (V > I) 236[1, 2] (I > T)	HLA class II	[10]
B6R	VACV -COP	*M. musculu s*	SKDVVQY EQEIESL EATYHI	261–280	SKDVVQY EQEIESL EATYHI		H2-d class II	[12]
B6R	VACV -COP	*H. sapiens*	GVIFLISVI VLVCSCD	289–304	GVIFLISII VLVCSCD	296 (V > I)	HLA class II	[8]

Ref. = reference epitope, VARV = variola virus, VACV-COP = vaccinia virus strain Copenhagen, VACV-WR = vaccinia virus strain Western Reserve, VACV-NYCBH-Dryvax = vaccinia virus strain New York City Board of Health, [1]only in VACV-WR, [2]only in VACV-COP, [3]only in MVA-BN

Supplementary Table 4. List of MPXV-derived peptide sequences used in this study.

This table lists all 81 synthesized peptides, each 20 amino acids long with 10 amino acid overlap. Peptides were derived from the MPXV proteins H3L (32 peptides), B6R (31 peptides), and A35R (18 peptides), covering the full length of each protein. Sequences correspond to Monkeypox virus isolate MPXV-M5312_HM12_Rivers. Peptides were synthesized by peptides&elephants GmbH (Hennigsdorf, Germany).

Name	N-Term	Sequence	C-Term	MW calc. g/mol	Weight theor.
H3L_aa1–20	H	MAAVKTPVIVVPVIDRPPSE	OH	2118.5	4.2 mg
H3L_aa11–30	H	VPVIDRPPSETFPNVHEHIN	OH	2297.4	4.6 mg
H3L_aa21–40	H	TFPNVHEHINDQKFDDVKDN	OH	2412.5	4.8 mg
H3L_aa31–50	H	DQKFDDVKDNEVMQEKRDVV	OH	2437.6	4.9 mg
H3L_aa41–60	H	EVMQEKRDVVIVNDDPDHYK	OH	2429.6	4.9 mg
H3L_aa51–70	H	IVNDDPDHYKDYVFIQWTGG	OH	2382.7	4.8 mg
H3L_aa61–80	H	DYVFIQWTGGNIRDDDKYTH	OH	2443.8	4.9 mg
H3L_aa71–90	H	NIRDDDKYTHFFSGFCNTMC	OH	2414.8	4.8 mg
H3L_aa81–100	H	FFSGFCNTMCTEETKRNIAR	OH	2355.8	4.7 mg
H3L_aa91–110	H	TEETKRNIARHLALWDSKFF	OH	2463.0	4.9 mg
H3L_aa101–120	H	HLALWDSKFFTELENKNVEY	OH	2483.9	5.0 mg
H3L_aa111–130	H	TELENKNVEYVVIIENDNVI	OH	2347.6	4.7 mg
H3L_aa121–140	H	VVIIENDNVIEDITFLRPVL	OH	2311.8	4.6 mg
H3L_aa131–150	H	EDITFLRPVLKAIHDKKIDI	OH	2365.1	4.7 mg
H3L_aa141–160	H	KAIHDKKIDILQMREIITGN	OH	2337.1	4.7 mg
H3L_aa151–170	H	LQMREIITGNKVKTELVIDK	OH	2329.0	4.7 mg
H3L_aa161–180	H	KVKTELVIDKDHAIFTYTGG	OH	2235.8	4.5 mg
H3L_aa171–190	H	DHAIFTYTGGYDVSLSAYII	OH	2206.8	4.4 mg
H3L_aa181–200	H	YDVSLSAYIIRVTTALNIVD	OH	2226.8	4.5 mg
H3L_aa191–210	H	RVTTALNIVDEIIKSGGLSS	OH	2073.7	4.1 mg
H3L_aa201–220	H	EIIKSGGLSSGFYFEIARIE	OH	2217.0	4.4 mg
H3L_aa211–230	H	GFYFEIARIENEMKINRQIM	OH	2503.2	5.0 mg
H3L_aa221–240	H	NEMKINRQIMDNSAKYVEHD	OH	2435.8	4.9 mg
H3L_aa231–250	H	DNSAKYVEHDPRLVAEHRFE	OH	2412.6	4.8 mg
H3L_aa241–260	H	PRLVAEHRFETMKPNFWSRI	OH	2515.0	5.0 mg
H3L_aa251–270	H	TMKPNFWSRIGTVAAKRYPG	OH	2280.9	4.6 mg
H3L_aa261–280	H	GTVAAKRYPGVMYTFTTPLI	OH	2186.8	4.4 mg
H3L_aa271–290	H	VMYTFTTPLISFFGLFDINV	OH	2326.0	4.7 mg
H3L_aa281–300	H	SFFGLFDINVIGLIVILFIM	OH	2272.3	4.5 mg
H3L_aa291–310	H	IGLIVILFIMFMLIFNVKSK	OH	2340.5	4.7 mg
H3L_301–320	H	FMLIFNVKSKLLWFLTGTFV	OH	2405.3	4.8 mg
H3L_aa305–324	H	FNVKSKLLWFLTGTFVTAFI	OH	2333.1	4.7 mg

B6R_aa1–20	H	MKTISVVTLLCVLPAVVYST	OH	2137.7	4.3 mg
B6R_aa11–30	H	CVLPAVVYSTCTVPTMNNAK	OH	2111.4	4.2 mg
B6R_aa21–40	H	CTVPTMNNAKLTSTETSFND	OH	2174.4	4.3 mg
B6R_aa31–50	H	LTSTETSFNDKQKVTFTCDS	OH	2252.5	4.5 mg
B6R_aa41–60	H	KQKVTFTCDSGYHSLDPNAV	OH	2210.5	4.4 mg
B6R_aa51–70	H	GYHSLDPNAVCETDKWKYEN	OH	2369.6	4.7 mg
B6R_aa61–80	H	CETDKWKYENPCKKMCTVSD	OH	2408.7	4.8 mg
B6R_aa71–90	H	PCKKMCTVSDYVSELYDKPL	OH	2319.8	4.6 mg
B6R_aa81–100	H	YVSELYDKPLYEVNSTMTLS	OH	2352.8	4.7 mg
B6R_aa91–110	H	YEVNSTMTLSCNGETKYFRC	OH	2346.7	4.7 mg
B6R_aa101–120	H	CNGETKYFRCEEKNGNTSWN	OH	2380.6	4.8 mg
B6R_aa111–130	H	EEKNGNTSWNDTVTCPNAEC	OH	2212.2	4.4 mg
B6R_aa121–140	H	DTVTCPNAECQPLQLEHGSC	OH	2145.2	4.3 mg
B6R_aa131–150	H	QPLQLEHGSCQPVKEKYSFG	OH	2275.6	4.6 mg
B6R_aa141–160	H	QPVKEKYSFGEYMTINCDVG	OH	2308.7	4.6 mg
B6R_aa151–170	H	EYMTINCDVGYEVIGVSYIS	OH	2255.7	4.5 mg
B6R_aa161–180	H	YEVIGVSYISCTANSWNVIP	OH	2215.6	4.4 mg
B6R_aa171–190	H	CTANSWNVIPSCQQKCDIPS	OH	2194.4	4.4 mg
B6R_aa181–200	H	SCQQKCDIPSLSNGLISGST	OH	2038.5	4.1 mg
B6R_aa191–210	H	LSNGLISGSTFSIGGVIHLS	OH	1959.7	3.9 mg
B6R_aa201–220	H	FSIGGVIHLSCKSGFTLTGS	OH	2011.7	4.0 mg
B6R_aa211–230	H	CKSGFTLTGSPSSTCIDGKW	OH	2075.6	4.2 mg
B6R_aa221–240	H	PSSTCIDGKWNPILPTCVRS	OH	2174.6	4.3 mg
B6R_aa231–250	H	NPILPTCVRSNEEFDPVDDG	OH	2217.4	4.4 mg
B6R_aa241–260	H	NEEFDPVDDGPDDETDLSKL	OH	2250.4	4.5 mg
B6R_aa251–270	H	PDDETDLSKLSKDVVQYEQE	OH	2338.5	4.7 mg
B6R_aa261–280	H	SKDVVQYEQEIESLEATYHI	OH	2381.6	4.8 mg
B6R_aa271–290	H	IESLEATYHIIIMALTIMGV	OH	2219.0	4.4 mg
B6R_aa281–300	H	IIMALTIMGVIFLISIIVLV	OH	2173.3	4.3 mg
B6R_aa291–310	H	IFLISIIVLVCSCDKNNDQY	OH	2300.9	4.6 mg
B6R_298–317	H	VLVCSCDKNNDQYKFHKLLP	OH	2364.8	4.7 mg
A35R_aa1–20	H	MMTPENDEEQTSVFSATVYG	OH	2236.4	4.5 mg
A35R_aa11–30	H	TSVFSATVYGDKIQGKNKRK	OH	2227.8	4.5 mg
A35R_aa21–40	H	DKIQGKNKRKRVIGLCIRIS	OH	2326.2	4.7 mg
A35R_aa31–50	H	RVIGLCIRISMVISLLSMIT	OH	2219.2	4.4 mg
A35R_aa41–60	H	MVISLLSMITMSAFLIVRLN	OH	2253.2	4.5 mg
A35R_aa51–70	H	MSAFLIVRLNQCMSANKAAI	OH	2181.9	4.4 mg
A35R_aa61–80	H	QCMSANKAAITDSAVAVAAA	OH	1893.3	3.8 mg
A35R_aa71–90	H	TDSAVAVAAASSTHRKVVSS	OH	1944.2	3.9 mg
A35R_aa81–100	H	SSTHRKVVSSTTQYDHKESC	OH	2280.4	4.6 mg
A35R_aa91–110	H	TTQYDHKESCNGLYYQGSCY	OH	2360.6	4.7 mg
A35R_aa101–120	H	NGLYYQGSCYILHSDYKSFE	OH	2387.9	4.8 mg
A35R_aa111–130	H	ILHSDYKSFEDAKANCAAES	OH	2199.6	4.4 mg

A35R_aa121–140	H	DAKANCAAESSTLPNKSDVL	OH	2034.4	4.1 mg
A35R_aa131–150	H	STLPNKSDVLTTWLIDYVED	OH	2309.7	4.6 mg
A35R_aa141–160	H	TTWLIDYVEDTWGSDGNPIT	OH	2283.6	4.6 mg
A35R_aa151–170	H	TWGSDGNPITKTTSDYQDSD	OH	2188.4	4.4 mg
A35R_aa161–180	H	KTTSDYQDSDVSQEVRKYFC	OH	2399.6	4.8 mg
A35R_aa162–181	H	TTSDYQDSDVSQEVRKYFCT	OH	2372.5	4.7 mg

Calc. = calculated, Term = terminus, theor. = theoretical, MW = Molecular Weigth.

Supplementary Table 5. Healthy donor reference group for TCR landscape analysis.

	**Healthy donors (HDs, n = 38)**
**Age, mean (range)**	39 (19-61)
**Sex, %**	
Male	42
Female	58
**HIV status**	
Negative, n	38
Positive, n	0

Supplementary Table 6. COVID-19 reference group for TCR landscape analysis (first pandemic wave, mild/moderate disease).

**Sample**	**Sex**	**Age**	**Sampling**	**S1 IgG**	**S1 IgA**	**NCP IgG**	**Comorbidities**
HACO6-3	f	68	d242	na	na	na	Adrenocortical adenoma; aHT; hypothyroidism
HACO7-4	f	70	d27	0.05	8.65	7.82	aHT; cholelithiasis; T2DM; steatosis
HACO11-1	m	30	d37	2.24	1.17	0.52	Pulmonary embolism
HACO12-1	f	29	d31	1.89	0.62	1.25	Asthma; hypothyroidism
HACO13-1	f	46	d36	1.11	2.19	1.57	Rheumatoid arthritis
HACO14-1	m	50	d38	1.84	1.00	1.70	
HACO16-1	m	37	d38	6.34	2.66	5.71	aHT
HACO17-1	f	25	d39	5.23	2.00	4.56	
HACO18-1	m	33	d39	3.64	4.10	3.65	
HACO26-1	m	49	d45	2.47	3.40	5.08	Asthma
HACO27-1	m	26	d45	0.11	0.08	0.06	
HACO28-1	f	33	d52	2.23	3.49	1.06	
HACO29-1	m	34	d51	5.02	7.78	4.19	
HACO32-1	f	38	d35	5.69	5.48	8.95	
HACO32-3	f	38	d235	na	na	na	

HACO33-1	m	34	d39	4.82	3.72	5.02	
HACO33-3	m	34	d240	na	na	na	
HACO34-1	m	40	d39	1.76	1.91	2.41	
HACO34-3	m	40	d240	na	na	na	
HACO35-1	f	62	d47	8.78	9.24	6.82	
HACO35-4	f	62	d224	na	na	na	
HACO38-1	f	27	d50	4.37	1.11	5.57	
HACO38-4	f	27	d227	na	na	na	
HACO45-1	m	32	d37	0.10	0.07	0.05	
HACO48-1	m	41	d52	5.34	3.55	2.23	
HACO49-1	f	56	d38	6.18	4.98	9.08	
HACO49-5	f	56	d206	na	na	na	
HACO50-1	f	45	d41	1.58	0.97	2.83	
HACO51-1	m	43	d49	3.05	2.44	3.43	
HACO52-1	f	30	d49	10.62	2.86	5.11	
HACO53-1	m	18	d39	8.63	7.91	1.72	
HACO54-1	f	58	d46	11.59	9.47	5.62	T2DM
HACO54-5	f	58	d206	na	na	na	T2DM
HACO55-1	f	38	d41	3.58	0.99	4.17	
HACO56-1	f	58	d41	12.40	4.30	7.07	
HACO56-4	f	59	d213	na	na	na	
HACO57-1	m	60	d30	7.14	8.61	5.94	
HACO57-4	m	60	d202	na	na	na	
HACO59-1	m	43	d50	9.82	3.90	6.01	
HACO61-1	m	11	d47	9.95	8.91	3.85	
HACO62-1	m	8	d64	8.37	2.48	4.05	
HACO63-1	f	11	d65	8.85	4.32	4.25	
HACO64-1	f	28	d73	7.60	2.95	2.18	
HACO66-1	f	41	d80	4.05	1.31	0.67	
HACO66-4	f	41	d227	na	na	na	
HACO70-2	m	68	d23	8.68	15.05	12.73	aHT; asthma; cerebral infarction; coronary heart disease; T2DM; myocardial infarction; PAD
HACO78-2	f	36	d201	na	na	na	
HACO79-2	m	50	d204	na	na	na	Asthma
HACO93-1	m	55	d201	na	na	na	Non-hodgkin lymphoma

aHT = arterial hypertension, d = day, f = female, m = male, na = not available, PAD = peripheral artery disease, T2DM = Diabetes mellitus type 2; S1 (spike protein subunit 1) IgG and IgA were detected using Anti-SARS-CoV-2 ELISA IgG/IgA Kit, Euroimmun AG, Lübeck Germany (Cat#EI 2606-9601 G; Cat#EI 2606-9601 A); NCP (nucleocapsid protein) IgG was detected using Anti-SARS-CoV-2 NCP ELISA IgG Kit, Euroimmun AG, Lübeck Germany (Cat#EI 2606-9601-2 G). Antibody titers were quantified as sample to calibrator ratio and interpreted as negative (ratio <0.8), borderline (0.8 ≥ ratio <1.1), or positive (ratio ≥ 1.1).

Supplementary Table 7. HCV-infected reference group for TCR landscape analysis (sustained virological response, after direct-acting antiviral therapy).

Sample	Sex	Age	Sampling	HCV antiviral therapy (brand name, treatment duration)	HCV genotype	antiHCV status	antiHCV ab	Comorbidities
HCV55	m	55	w74	Ombitasvir/Paritaprevir/ Ritonavir, Dasabuvir (Viekirax, Exviera, 12w)	GT4a	positive	15	aHT; T2DM; s/p HBV infection
HCV56	f	57	w37	Ribavirin, Sofosbuvir (Sovaldi, 12w)	GT3a	positive	8	aHT; asthma bronchiale; cryoglobulinemic vasculitis
HCV57	m	57	w83	Pegylated IFNα/Ribavirin (12w)	GT1a	positive	15	aHT
HCV58	m	58	w15	Ledipasvir/Sofosbuvir (Harvoni, 8w)	GT1b	positive	>11.00	aHT
HCV54	f	65	w71	Ledipasvir/Sofosbuvir (Harvoni, 12w)	GT1b	positive	>11.00	aHT
HCV59	m	52	w147	Pegylated IFNα/Ribavirin (na)	GT1a	na	na	na
HCV60	f	39	w38	Ledipasvir/Sofosbuvir (12w)	GT1a	positive	>11.00	
HCV61	m	32	w158	Pegylated IFNα/Ribavirin (24w)	GT1b	positive	17	
HCV53	f	65	w60	Maviret (8w)	GT6	positive	>11.00	aHT
HCV52	f	56	w72	Velpatasvir/Sofosbuvir (Epclusa, 12w)	GT3	positive	14	
HCV51	f	53	w36	Velpatasvir/Sofosbuvir (Epclusa, 12w)	GT3a	positive	>11.00	
HCV50	f	56	w115	Velpatasvir/Sofosbuvir (Epclusa, 12w)	GT3	positive	>11.00	Rheumatoid arthritis (susp.); T2DM
HCV62	m	60	w29	Velpatasvir/Sofosbuvir (12w)	GT2	positive	>11.00	aHT
HCV63	f	53	w189	Ombitasvir/Paritaprevir/ Ritonavir (Viekirax, na)	GT1b	positive	>11.00	T2DM
HCV64	m	49	w29	Ledipasvir/Sofosbuvir (Harvoni, 8w)	GT1b	positive	>11.00	Hereditary factor VII deficiency; hypermenorrhea
HCV65	m	38	w39	Velpatasvir/Sofosbuvir (Epclusa, 12w)	GT3	positive	>11.00	
HCV66	m	41	w108	Ribavirin, Velpatasvir/Sofosbuvir (Epclusa, 12w)	GT3	positive	>11.00	
HCV67	m	39	w36	Velpatasvir/Sofosbuvir (Epclusa, 12w)	GT3	positive	>11.00	
HCV68	m	51	w130	Velpatasvir/Sofosbuvir (Epclusa, 12w)	GT3	positive	>11.00	

HCV49	f	54	w58	Velpatasvir/Sofosbuvir (Epclusa, 12w)	GT3	positiv e	>11.00	
HCV48	f	48	w41	Elbasvir/Grazoprevir (Zepatier, 12w)	GT1b	positiv e	>11.00	
HCV69	m	68	w65	Velpatasvir/Sofosbuvir (Epclusa, 12w)	GT3	positiv e	>11.00	aHT
HCV70	f	57	w22	Glecaprevir/Pibrentasvir (Maviret, 8w)	GT3a	positiv e	>11.00	
HCV71	m	77	w43	Sofosbuvir/Velpatasvir/ Voxilaprevir (Vosevi, 12w)	GT1b	positiv e	>11.00	aHT; T2DM
HCV72	f	39	w92	Velpatasvir/Sofosbuvir (Epclusa, 12w)	GT3	positiv e	>11.00	
HCV73	m	57	w40	Glecaprevir/Pibrentasvir (Maviret, 8w)	GT5	positiv e	>11.00	Rheumatoid arthritis; T2DM

ab = antibody(ies), aHT = arterial hypertension, f = female, IFNα = interferon α, m = male, na = not available, s/p = status post, susp. = suspected, T2DM = Diabetes mellitus type 2, w = week(s); AntiHCV antibodies were detected using the Alinity anti-HCV reagent kit against recombinant c100-3, HCr43 HCV antigens, Abbott GmbH, Wiesbaden Germany (Ref 06P0460), values are given as ratio sample to cutoff.

Supplementary References

1 Jing, L. *et al.* An extremely diverse CD4 response to vaccinia virus in humans is revealed by proteome-wide T-cell profiling. *J Virol* **82**, 7120-7134 (2008). https://doi.org/10.1128/JVI.00453-08

2 Jing, L. *et al.* CD4 T-cell memory responses to viral infections of humans show pronounced immunodominance independent of duration or viral persistence. *J Virol* **87**, 2617-2627 (2013). https://doi.org/10.1128/JVI.03047-12

3 Jing, L. *et al.* Dominance and diversity in the primary human CD4 T cell response to replication-competent vaccinia virus. *J Immunol* **178**, 6374-6386 (2007). https://doi.org/10.4049/jimmunol.178.10.6374

4 Moutaftsi, M. *et al.* Vaccinia virus-specific CD4+ T cell responses target a set of antigens largely distinct from those targeted by CD8+ T cell responses. *J Immunol* **178**, 6814-6820 (2007). https://doi.org/10.4049/jimmunol.178.11.6814

5 Sette, A. *et al.* Selective CD4+ T cell help for antibody responses to a large viral pathogen: deterministic linkage of specificities. *Immunity* **28**, 847-858 (2008). https://doi.org/10.1016/j.immuni.2008.04.018

6 Falivene, J. *et al.* Improving the MVA vaccine potential by deleting the viral gene coding for the IL-18 binding protein. *PLoS One* **7**, e32220 (2012). https://doi.org/10.1371/journal.pone.0032220

7 Siciliano, N. A. *et al.* Impact of distinct poxvirus infections on the specificities and functionalities of CD4+ T cell responses. *J Virol* **88**, 10078-10091 (2014). https://doi.org/10.1128/JVI.01150-14

8 Kennedy, R. B. & Poland, G. A. The identification of HLA class II-restricted T cell epitopes to vaccinia virus membrane proteins. *Virology* **408**, 232-240 (2010). https://doi.org/10.1016/j.virol.2010.09.013

9 Sirven, P., Castelli, F. A., Probst, A., Szely, N. & Maillere, B. In vitro human CD4+ T cell response to the vaccinia protective antigens B5R and A33R. *Mol Immunol* **46**, 1481-1487 (2009). https://doi.org/10.1016/j.molimm.2008.12.016

10 Tang, J. *et al.* Human T-cell responses to vaccinia virus envelope proteins. *J Virol* **80**, 10010-10020 (2006). https://doi.org/10.1128/JVI.00601-06

11 Thiele, F. *et al.* Modified vaccinia virus Ankara-infected dendritic cells present CD4+ T-cell epitopes by endogenous major histocompatibility complex class II presentation pathways. *J Virol* **89**, 2698-2709 (2015). https://doi.org/10.1128/JVI.03244-14

12 Berhanu, A. *et al.* Vaccination of BALB/c mice with Escherichia coli-expressed vaccinia virus proteins A27L, B5R, and D8L protects mice from lethal vaccinia virus challenge. *J Virol* **82**, 3517-3529 (2008). https://doi.org/10.1128/JVI.01854-07
